# Scutellarein ameliorates dextran sulfate sodium-induced ulcerative colitis by inhibiting colonic epithelial cell proinflammation and barrier disruption

**DOI:** 10.3389/fphar.2024.1479441

**Published:** 2024-10-21

**Authors:** Qinglian Tang, Haidong Jia, Xu Qin, Zhaowen Lu, Wenjie Huang, Yujing Wang, Zhengyu Cao

**Affiliations:** ^1^ Jiangsu Provincial Key Laboratory for TCM Evaluation and Translational Research, School of Traditional Chinese Pharmacy, China Pharmaceutical University, Nanjing, Jiangsu, China; ^2^ R&D Center, Shanghai Jahwa United Co., Ltd., Shanghai, China

**Keywords:** scutellarein, ulcerative colitis, epithelial barrier integrity, NF-κB, proinflammation

## Abstract

**Introduction:**

Scutellarein (Scu) is a natural occurring flavonoid found in multiple traditional Chinese medicines such as *Oroxylum* indicum (L.) Kurz and *Scutellaria baicalensis*, with various pharmacological activities including anti-inflammation, anti-oxidation and myocardial protection. Here, we investigated the therapeutic efficacy of Scu on ulcerative colitis (UC) and the underlying mechanism.

**Methods:**

Efficacy of Scu on UC was evaluated in dextran sulfate sodium (DSS) induced colitis mouse model. Inflammation in colonic tissues was assessed by myeloperoxidase activity assay and RT-qPCR. Barrier proteins expression was examined using immunostaining and Western blot. IL-1β-treated HT-29 cells was used for mechanical investigation.

**Results:**

Gavage of Scu significantly decreased the DAI score, improved colon shortening, ameliorated the pathological score in DSS-treated mice with better efficacy than the positive drug, 5-aminosalicylic acid. Scu also inhibited the expression levels of cytokines (*Il-1β*, *Tnf-α*, *Il-1α*, *Il-6*, and *Cxcl1*) as well as barrier proteins (E-cadherin, Occludin, and ZO-1) in colon tissues of DSS mice. In intestinal epithelial HT-29 cells, Scu attenuated the IL-1β-downregulated expression levels of E-cadherin, occludin, and ZO-1, while reduced IL-1β-upregulated *IL-6* and *IL-8* mRNA levels. Moreover, Scu inhibited the phosphorylation and nuclear translocation of NF-κB and suppression of NF-κB phosphorylation abolished IL-1β-disrupted epithelial barrier integrity and IL-1β-upregulated proinflammatory mediators expression in HT-29 cells.

**Conclusion:**

These data demonstrate that Scu is an efficacious therapeutic agent to treat UC. Inhibition of inflammatory responses and maintenance of epithelial barrier integrity through NF-κB signaling pathway underlines Scu therapeutic effect on UC.

## Introduction

Ulcerative colitis (UC) is a common chronic inflammatory bowel disease (IBD) that is characterized by abdominal pain, diarrhea, and hematochezia (Ungaro et al., 2017; Anbazhagan et al., 2018). UC affects approximately 5 million people worldwide, and the incidence is increasing (Le Berre et al., 2023). Despite the availability of current treatments such as 5-aminosalicylic acid (5-ASA), steroids, immunosuppressants, and biologics, approximately 20% of patients with refractory remission-relapsing symptoms still require proctocolectomy (Le Berre et al., 2023).

Although the etiology is not fully understood, genetic susceptibility, gut barrier defects, dysbiosis, and dysregulated immune responses and their interplays underpin the initiation and progression of UC (Ramos and Papadakis, 2019). The intestinal epithelial barrier not only selectively regulates barrier permeability for ion/nutrient/water absorption but also prevents the entry of external harmful pathogens/toxins (Di Tommaso et al., 2021). To preserve the integrity of the intestinal barrier, the expression, distribution, and fine balance between isoforms of tight junction proteins, including claudins, occludins, and zonula occludens (ZO) family, must be tightly regulated (Chelakkot et al., 2018). Evidence has demonstrated that a variety of stimuli, such as tumor necrosis factor-α (TNF-α) and interleukin-1β (IL-1β), regulate the expression and organization of tight junction proteins in both cellular models and *in vivo* pathological conditions (Ye et al., 2006; Turner, 2009; Su et al., 2013; Lee et al., 2024). The disruption of the intestinal barrier and loss of mucosal homeostasis have been observed in active UC patients and shown to trigger intestinal inflammation (Van der Post et al., 2019). Although UC is an immune disease, the gut homeostasis and function coordinate immune defense and bacterium-immune cell crosstalk. It is accepted that the prevention of the dysregulation of the gut barrier may represent a strategy to prevent the progression of UC. One example is Anakinra, an IL-1R inhibitor used for IBD therapy, which has been launched in a phase II clinical trial for severe UC (Shouval et al., 2016; Thomas et al., 2019).

Scutellarein (Scu) is a naturally occurring flavonoid found in multiple traditional Chinese medicines such as *Oroxylum* indicum (L.) Kurz, *Erigeron* breviscapus (Vaniot) Hand.-Mazz, and *Scutellaria* baicalensis. These traditional Chinese herbs have anti-inflammatory, anti-viral, and anti-cancer properties (Dinda et al., 2015; Zhao et al., 2019; Wu et al., 2021) and have been utilized to treat cardiovascular, neurological, cutaneous inflammatory diseases, respiratory tract infections, and liver inflammation (Zhao et al., 2019; Liao et al., 2021). Huangqin Decoction has been shown to ameliorate dextran sodium sulfate (DSS)-induced UC in mice, possibly by suppressing intestinal barrier disruption (Li M. et al., 2022; Mo et al., 2022). As one of the major active flavonoids in these Chinese herbs, the anti-inflammatory effects of Scu have been demonstrated in numerous studies. Scu has been found to suppress lipopolysaccharide (LPS)-induced inflammation in RAW264.7 macrophages (Park et al., 2022), relieve OGD/R-induced cell death and proinflammation of tubular epithelial cells (Liu et al., 2021), reduce IL-1β-induced apoptosis and senescence in rat chondrocytes (Jing et al., 2024), and inhibit the microglia BV2 cell activation (Talavera et al., 2020). We have also demonstrated that Scu alleviates 2,4-dinitrofluorobenzene (DNFB) and carvacrol-induced pruritus and dermatitis by specifically inhibiting transient receptor potential vanilloid 3 (TRPV3) channels (Wang et al., 2022). Apart from its anti-inflammatory effect, Scu also displays activities of anti-proliferation, anti-apoptosis, neuroprotection, and anti-cancer (Shi et al., 2015; Wang et al., 2022; Rizou et al., 2023; Thoa and Son, 2024).

In the present study, we investigated the impact of Scu on colonic inflammation in the UC mouse model induced by DSS. We demonstrate Scu as an effective therapeutic agent to treat UC with better efficacy than 5-ASA, the positive drug. Mechanistic exploration demonstrates that Scu inhibits inflammatory responses and preserves barrier integrity by inhibiting the NF-κB signaling pathway.

## Materials and methods

### Materials

Dulbecco’s modified Eagle medium (DMEM), fetal bovine serum (FBS), penicillin/streptomycin (P/S), and trypsin were purchased from Life Technology (Grand Island, NY, United States). Scutellarein (Scu, Cat# B21479), with a purity above 98%, was obtained from Earay Bio-tech (Baoji, Shanxi China). Dextran sulfate sodium (DSS, with the highest sulfur content of 19% and a molecular weight ranging from 36 to 50 kDa, Cat# 160110) was obtained from MP Biomedicals (Santa Ana, CA, United States). BAY11-7085 (Cat# HY-10257), 5-aminosalicylic acid (5-ASA, Cat# HY-15027), and IL-1β (Cat# HY-P78459A) were obtained from MedChemExpress (Monmouth Junction, NJ, United States). The Myeloperoxidase (MPO) Activity Assay Kit (Cat# A114-1–1) was supplied by the Nanjing Jiancheng Bioengineering Institute (Nanjing, Jiangsu, China). Anti-ZO-1 antibody (Cat# 21773-1-AP) was acquired from Proteintech (Wuhan, Hubei, China). The following antibodies were sourced from Cell Signaling Technology (Danvers, MA, United States): antibodies against phosphor (p)-NF-κB p65 (Cat# 3033), NF-κB (Cat# 8242), occludin (Cat# 91131), E-cadherin (Cat# 3195), and anti-rabbit Alexa Fluor^®^ 488 (Cat# 4412). The primary antibody against GAPDH (Cat# MB001) was purchased from Bioworld Biotechnology Co., Ltd. (Nanjing, Jiangsu, China). Tribromoethanol, Hoechst 33342, and all the inorganic salts were supplied by Sigma-Aldrich (St. Louis, MO, United States) unless otherwise mentioned. HT-29 cells (Cat# HTB-38) were procured from the Cell Bank of Shanghai (Shanghai, China).

### Animals and ethics statement

Male C57BL/6 mice, aged 7–8 weeks and weighing between 22 and 24 g, were sourced from Yangzhou University (Yangzhou, Jiangsu, China). The animal care and usage were conducted following the guidelines established by the National Institutes of Health, as detailed in NIH Publications No. 8023 (revised in 1978). All experimental procedures involving animals adhered to ethical standards for animal research and received approval from the Ethics Committee of the China Pharmaceutical University (#SYXK, 2023–0019). The mice were housed in a vivarium with a controlled temperature of 23°C ± 2°C, under a 12-hour light and dark cycle, and had unrestricted access to food and water.

### DSS-induced UC and drug administration

A UC mouse model was established following the methodology described in previous research (Xue et al., 2023). In brief, a total of 42 mice were acclimatized for 1 week and then randomly assigned to 7 different experimental groups. To induce colonic inflammation, mice were challenged with a 3% concentration of DSS in their drinking water for seven consecutive days. Mice in the vehicle (Veh) group and 20 mg/kg Scu group were provided with regular drinking water.

Scu and 5-ASA were dissolved in a solution comprising 2% dimethyl sulfoxide (DMSO), 2% Tween-80, and 0.5% sodium carboxymethyl cellulose (CMC-Na). A measure of 200 mg/kg 5-ASA and Scu at varying levels of 5 mg/kg, 10 mg/kg, and 20 mg/kg were administered to the mice once daily by intragastric gavage for seven consecutive days from the first day of DSS administration until the day of sacrifice by tribromoethanol administration. The distal colon tissues were carefully dissected, harvested, and immediately frozen for further analysis.

### Disease activity index score

The disease activity index (DAI) was scored according to Sann’s method by summing the respective scores for body weight loss, hemorrhage, and faecal consistency (Sann et al., 2013; Wang et al., 2021).

### Myeloperoxidase activity assay

The enzymatic activity of MPO in mouse colon samples was evaluated using an MPO activity assay kit (Alkushi et al., 2022). According to the manufacturer’s instructions, the colonic homogenate supernatant reacts with H_2_O_2_ and o-dianisidine to produce oxidized o-dianisidine, the absorbance of which at 460 nm is linearly correlated with MPO activity. The MPO results were expressed as units per gram (U/g) of tissue wet weight.

### Hematoxylin and eosin (H&E) staining

Colon samples were fixed in 4% paraformaldehyde, paraffin-embedded, and sectioned at 5 μm, followed by staining with hematoxylin and eosin. The sections were photographed using the NanoZoomer 2.0 RS Slide scanner (Hamamatsu Photonics, Japan). According to Wang’s method (Wang et al., 2018), histological scores were assessed by summing the respective scores for inflammation, the extent of crypt damage, and the range of lesions.

### Cell culture

The HT-29 cells, human intestinal epithelial cells, were maintained in DMEM supplemented with 10% FBS and 100 U/mL of P/S at 37°C with 5% CO_2_.

### Western blotting

Western blotting was performed, as previously described (Li S. H. et al., 2022). In brief, 30 µg of protein samples were subjected to sodium dodecyl sulfate-polyacrylamide gel electrophoresis (SDS-PAGE) for separation, followed by transfer to a nitrocellulose membrane. The membranes were blocked by 5% skimmed milk and then incubated with primary antibodies: anti-ZO-1, anti-p-NF-κB p65, anti-NF-κB, anti-occludin, anti-E-cadherin, anti-β-actin, and anti-GAPDH (at the recommended dilution). After the incubation with IRDye (680RD or 800CW)-labeled goat anti-mouse or goat anti-rabbit secondary antibodies (at 1:10,000 dilution), the blots were visualized using the LI-COR Odyssey Infrared Imaging System (LI-COR Biosciences, Lincoln, NE, United States), and the densitometry was quantified using its application software (version 2.1). The expression levels of proteins were normalized to those of β-actin or GAPDH.

### Real-time-quantitative polymerase chain reaction analysis

Real-time quantitative polymerase chain reaction (RT-qPCR) was performed, as previously described (Wang et al., 2022). Total messenger RNA (mRNA) of colon tissues or HT-29 cells was extracted using TRIzol reagent (Cat# R401-01, Vazyme) and subsequently reverse-transcripted to cDNA using the HiScript II Q RT SuperMix Kit (Cat# R223-01, Vazyme). RT-qPCR was conducted on a QuantStudio 3 System (Thermo Fisher Scientific, Massachusetts, United States). Relative mRNA expression levels normalized to GAPDH were quantified using the 2^−ΔΔCt^ method. All primers listed in Table 1 were synthesized by Beijing Tsingke Biotechnology Co., Ltd. (Beijing, China).

**TABLE 1:** Primers used in this study.

Gene	Sense (5′−3′)	Anti-sense ( 5′−3′)
*mGAPDH*	CAT​CTT​CCA​GGA​GCG​AGA​CC	GAA​GGG​GCG​GAG​ATG​ATG​AC
*mTnf-α*	GAC​GTG​GAA​CTG​GCA​GAA​GAG	TGC​CAC​AAG​CAG​GAA​TGA​GA
*mCxcl1*	GCT​GGG​ATT​CAC​CTC​AAG​AA	CTT​GGG​GAC​ACC​TTT​TAG​CA
*mIl-6*	GTT​CTC​TGG​GAA​ATC​GTG​GA	TTC​TGC​AAG​TGC​ATC​ATC​GT
*mIl-1α*	CGA​AGA​CTA​CAG​TTC​TGC​CAT​T	GAC​GTT​TCA​GAG​GTT​CTC​AGA​G
*mIl-1β*	GAA​ATG​CCA​CCT​TTT​GAC​AGT​G	TGG​ATG​CTC​TCA​TCA​GGA​CAG
*hGAPDH*	AAC​GGA​TTT​GGT​CGT​ATT​GGG	TCG​CTC​CTG​GAA​GAT​GGT​GAT
*hIL-6*	GTG​TGA​AAG​CAG​CAA​AGA​G	CTC​CAA​AAG​ACC​AGT​GAT​G
*hIL-8*	GTC​CTT​GTT​CCA​CTG​TGC​CT	GCT​TCC​ACA​TGT​CCT​CAC​AA

### Transepithelial electrical resistance measurement

As previously described (Srinivasan et al., 2015), HT-29 cells were seeded in the upper chamber, and the transepithelial electrical resistance (TEER) was recorded using Millcell®ERS (Electrical Resistance System) with two electrodes placed in the upper and lower chambers, respectively. The TEER values were reported in units of ohm-centimeter squared (Ω·cm^2^).

### Immunofluorescence labeling

Colon slides were subject to deparaffinization and subsequent antigen retrieval. HT-29 cells were seeded in a confocal petri dish and fixed with 4% paraformaldehyde. After permeabilization using 0.3% Triton X-100, colon slides and HT-29 cells were blocked with 5% BSA at room temperature for 1 h, followed by incubation overnight with primary antibodies against ZO-1 (1:1,200), occludin (1:400), E-cadherin (1:1,000), and NF-κB (1:400), respectively. The goat anti-mouse or anti-rabbit secondary antibodies conjugated with Alexa Fluor^®^ 488 were used for visualization. Nuclei were stained with Hoechst. Slides and cells were photographed using a Zeiss LSM 800 Confocal Fluorescence Microscope (Le Pecq, France) with a 20 × (slides) or 63 × (cells) objective.

### Cell viability assay

As previously described (Xue et al., 2023), HT-29 cells seeded in 96-well plates were treated with Scu at various concentrations for 24 h. After the replacement with the fresh medium containing 500 μg/mL of MTT, a further incubation was carried out for 1 h. Then, the medium was replaced by DMSO to dissolve the formazan crystals. Absorbance at 570 nm was measured using an Infinite M200 Pro-NanoQuant Reader from Tecan Austria (GmbH, Grodig, Austria).

### Statistical analysis

Statistical analysis was performed using GraphPad Prism version 8.01 (San Diego, CA, United States). Data are presented as the mean ± SEM. Statistical significance was assessed using one-way ANOVA or two-way ANOVA, followed by Bonferroni’s multiple comparison test. A *p*-value less than 0.05 was deemed statistically significant.

## Results

### Scu ameliorated DSS-induced UC in mice

**FIGURE 1:**
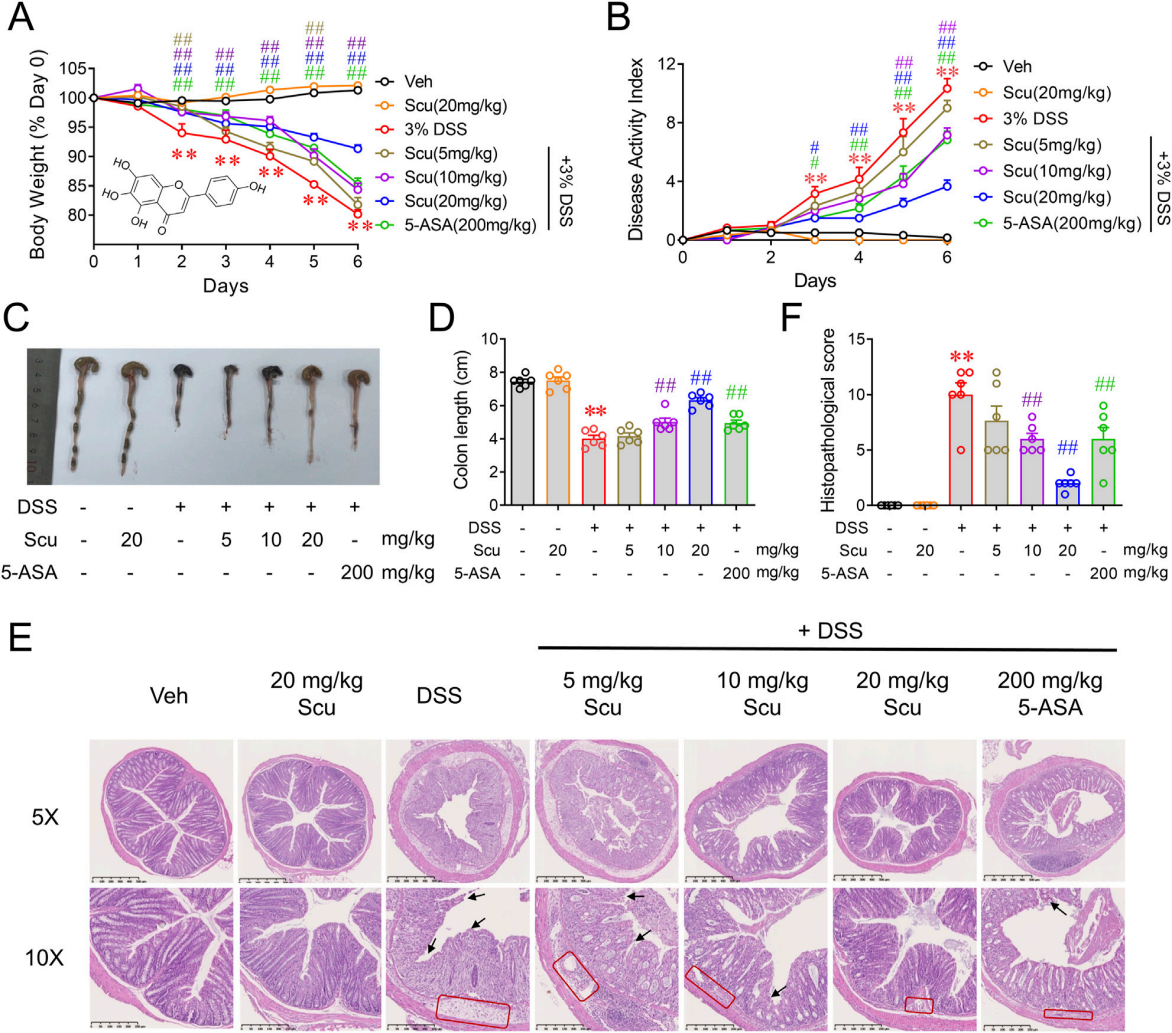
Scu ameliorated DSS-induced ulcerative colitis in mice. **(A)** Changes in body weight in mice treated with vehicle or DSS, administered with Scu and 5-ASA for 7 days. Inset: structure of Scu. **(B)** Disease activity index of mice treated with vehicle and DSS, administered with Scu and 5-ASA. **(C)** Representative colon images and **(D)** quantification of colon length in vehicle and DSS-treated mice administered with Scu and 5-ASA. **(E)** Representative micrographs of colon tissues from mice in different groups stained with H&E. Disappearance of crypts and edematous lamina propria containing inflammatory cell infiltrates are labeled by black arrows and red boxes, respectively. Scale bars are 500 μm in the upper panel images (5 × magnification) and 250 μm in the lower panel images (10 × magnification). **(F)** Quantification of histopathological scores from H&E-stained colon tissues from mice in different groups. Data are expressed as the mean ± SEM. N = 6 mice; **, *p* < 0.01 vs. Veh; ^#^, *p* < 0.05, ^##^, *p* < 0.01, vs. DSS.

The structure of Scu is illustrated in Figure 1A inset. Consecutive drinking of 3% DSS led to a gradual reduction in the body weight of the mice (Figure 1A). The administration of Scu alone did not affect the mouse body weight and dose-dependently alleviated the body weight loss induced by DSS (Figure 1A). In addition to body weight loss, DSS also induced diarrhea and bloody stools. These symptoms can be quantified using the disease activity index (DAI) score. The DAI scores were significantly increased on day 3 and gradually increased upon drinking of DSS. The administration of Scu also dose-dependently suppressed the DSS-induced severity of UC, as reflected by the decreased DAI scores. At 20 mg/kg, Scu displayed greater protection against DSS-induced UC than the positive control, 5-ASA (Figure 1B). Compared to the Veh group, mice with DSS administration exhibited shortened colon length (7.45 ± 0.14 cm vs. 4.02 ± 0.20 cm, *p* < 0.01) (Figures 1C, D). While 5 mg/kg Scu showed marginal improvement (4.02 ± 0.20 cm vs. 4.17 ± 0.19 cm, *p* > 0.01) in DSS-induced colon atrophy, doses of 10 mg/kg, 20 mg/kg Scu, and 200 mg/kg 5-ASA were able to diminish DSS-induced colon atrophy by 29.1% (*p* < 0.01), 67.0% (*p* < 0.01), and 27.1% (*p* < 0.01), respectively (Figures 1C, D). Pathological examination indicated that colon tissues from DSS mice experienced destruction with the disappearance of crypts (black arrows) and edematous lamina propria containing inflammatory cell infiltrates (red boxes). The administration of Scu and 5-ASA preserved the epithelial barrier, reduced edema, and alleviated inflammatory infiltration in the lamina propria of DSS mice (Figure 1E). The pathology score of DSS mice was 10.0 ± 1.1 (*p* < 0.01 vs. control). While oral administration of 20 mg/kg Scu alone did not impact colon pathology, doses of 10 mg/kg and 20 mg/kg Scu significantly decreased the pathological scores in DSS mice to 6.0 ± 0.5 (*p* < 0.01) and 2.0 ± 0.3 (*p* < 0.01), respectively. As the positive drug, 5-ASA also reduced the pathological score to 6.0 ± 1.0 (*p* < 0.01) (Figure 1F). These findings indicated that Scu yielded superior protection on UC when compared to the clinically used drug, 5-ASA.

### Scu attenuated the inflammation in DSS-treated mice

**FIGURE 2:**
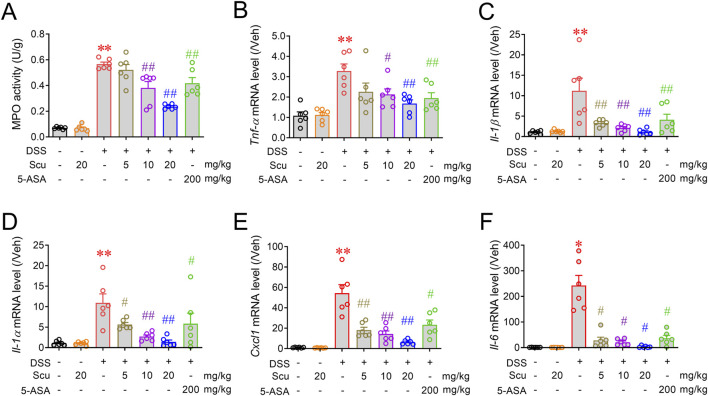
Scu attenuated colonic inflammation in DSS-treated mice. **(A)** MPO activity in colon tissues from Veh- and DSS-treated mice, administered with Scu and 5-ASA. **(B–F)** Quantitative RT-PCR analysis of mRNA levels of *Tnf-α* **(B)**, *Il-1β* **(C)**, *Il-1α* **(D)**, *Cxcl1* **(E)**, and *Il-6* **(F)**, respectively, in colon tissues from different groups of mice. Data are presented as the mean ± SEM. N = 6 mice; **, *p* < 0.01 vs. Veh; ^#^, *p* < 0.05, ^##^, *p* < 0.01 vs. DSS. One-way ANOVA with Bonferroni’s multiple comparisons tests.

Given the superior protection of Scu on UC in DSS-treated mice, we next evaluated the impact of Scu on the inflammatory response in colonic tissues. DSS-administered mice displayed increased MPO activity in colonic tissues and upregulated mRNA expression levels of inflammatory cytokines such as *Tnf-α*, *Il-1β*, *Il-1α*, *Cxcl1*, and *Il-6* (Figure 2). Although oral administration of 20 mg/kg Scu alone did not affect the MPO activity, Scu dose-dependently reduced the MPO activity and the mRNA expression levels of these inflammatory mediators (Figure 2). At the dose of 20 mg/kg, Scu significantly decreased the MPO activity by 66.6% ± 1.6% (*p* < 0.01), while 5-ASA only achieved 29.8% ± 8.8% reduction (*p* < 0.01). Scu at 20 mg/kg also remarkably decreased mRNA expression levels of *Tnf-α*, *Il-1β*, *Il-1α*, *Cxcl1*, and *Il-6* by 72.8% ± 8.5% (*p* < 0.01), 99.4% ± 2.9% (*p* < 0.01), 96.9% ± 4.6% (*p* < 0.01), 90.0% ± 1.8% (*p* < 0.01), and 99.0% ± 0.6% (*p* < 0.01), respectively, in the colons of DSS-treated mice (Figure 2). As a positive drug, 5-ASA also significantly reduced mRNA expression levels of *Tnf-α*, *Il-1β*, *Il-1α*, *Cxcl1*, and *Il-6* by 60.5% ± 11.9% (*p* < 0.01), 70.0% ± 13.5% (*p* < 0.01), 51.8% ± 26.2% (*p* < 0.01), 58.1% ± 8.7% (*p* < 0.01), and 85.1% ± 4.5% (*p* < 0.01), respectively, in the colons of DSS-treated mice (Figure 2). Again, Scu displayed better suppression on colonic inflammation in the DSS-treated mice than 5-ASA.

### Scu ameliorated damage of the epithelial barrier in DSS mice

**FIGURE 3:**
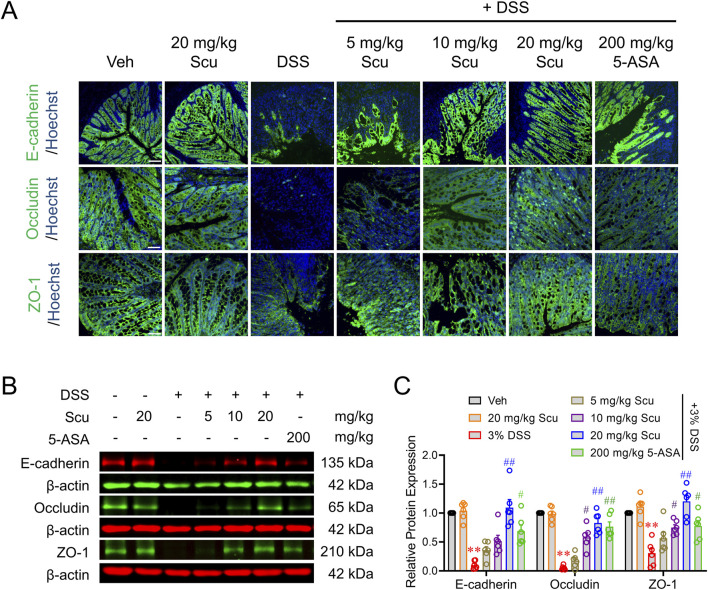
Scu increased the barrier proteins expression in DSS mice. **(A)** Representative immunofluorescent images of E-cadherin (upper panel), occludin (middle panel), and ZO-1 (lower panel) labeled colon tissues from Veh- and DSS-treated mice, administered with Scu and 5-ASA. Nuclei were stained with Hoechst 33342. Scale bar = 50 μm. **(B)** Representative Western blots of E-cadherin, occludin, and ZO-1 in colon tissues from mice with different drug administrations. **(C)** Quantification of E-cadherin, occludin, and ZO-1 protein expression in colon tissues from mice with different drug administrations. Data are expressed as the mean ± SEM. N = 6 mice; **, *p* < 0.01 vs. Veh; ^#^, *p* < 0.05, ^##^, *p* < 0.01 vs. DSS. One-way ANOVA with Bonferroni’s multiple comparisons tests.

The disruption of the gut barrier and loss of mucosal homeostasis were observed in active UC patients and shown to trigger intestinal inflammation (Van der Post et al., 2019). To maintain the integrity of the intestinal barrier, the expression, distribution, and fine balance between isoforms of tight junction proteins, including occludins, claudins, and ZO family, must be tightly regulated (Chelakkot et al., 2018). We, therefore, investigated the impact of Scu on the expression of tight junction proteins in DSS-treated mice. Immunofluorescence staining demonstrated that E-cadherin, occludin, and ZO-1 were highly expressed in the crypts of colons in vehicle-treated mice. The administration of Scu (20 mg/kg) alone did not change the expression levels and patterns of E-cadherin, occludin, and ZO-1. However, in the colons of DSS-treated mice, the expressions of E-cadherin, occludin, and ZO-1 were all diminished. The administration of Scu dose-dependently alleviated the downregulated protein expression of E-cadherin, occludin, and ZO-1, a phenomenon also observed by 5-ASA treatment (Figure 3A). Consistent with the immunofluorescence measurement, Western blots also demonstrated that DSS-treatment decreased the protein levels of E-cadherin, occludin, and ZO-1 by 89.8% ± 2.9% (*p* < 0.01), 94.8% ± 1.4% (*p* < 0.01), and 69.5% ± 8.6% (*p* < 0.01), respectively. The administration of Scu dose-dependently reversed the protein expression of E-cadherin, occludin, and ZO-1 in DSS mice. At 20 mg/kg, Scu increased the expression levels of E-cadherin, occludin, and ZO-1 in DSS-treated mice from 10.2% ± 2.9% to 109.2% ± 15.7% (*p* < 0.01), 5.2% ± 1.4% to 82.8% ± 10.6% (*p* < 0.01), and 30.5% ± 8.6% to 120.2% ± 25.3% (*p* < 0.01) of vehicle-treated mice, respectively (Figures 3B, C).

### Scu inhibited IL-1β-induced epithelial inflammation and barrier damage in HT-29 cells

**FIGURE 4:**
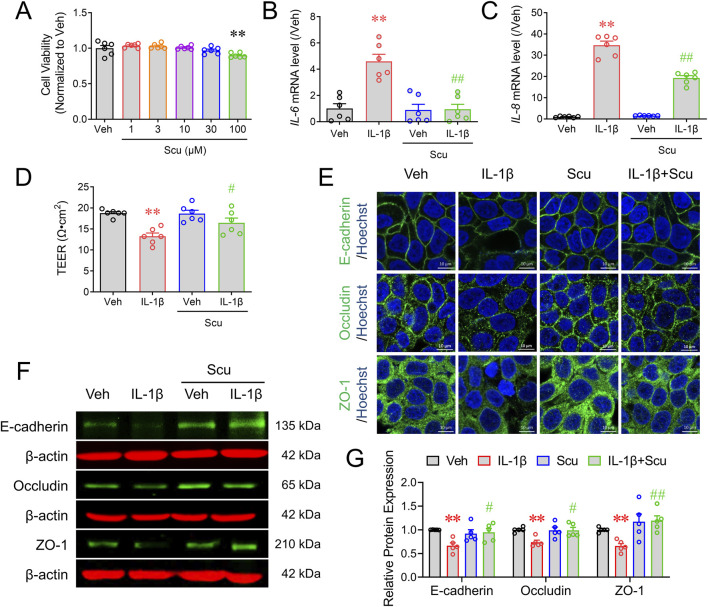
Scu inhibited IL-1β-induced mRNA expression of inflammatory mediators and barrier damage of HT-29 cells. **(A)** HT-29 cell viability measured by the MTT assay after being exposed to different concentrations of Scu for 24 h **(B, C)** Quantitative RT-PCR analysis of mRNA levels of *IL-6* **(B)** and *IL-8* **(C)** in HT-29 cells treated with IL-1β (10 ng/mL) for 12 h in the absence and presence of Scu (10 μM). **(D)** Change in the TEER of HT-29 monolayer treated with IL-1β (10 ng/mL, 24 h) in the absence and presence of Scu (10 μM). **(E)** Representative immunofluorescence images of E-cadherin (upper panel), occludin (middle panel), and ZO-1 (lower panel) in HT-29 cells after being exposed to IL-1β (10 ng/mL) for 24 h in the absence and presence of Scu (10 μM). Nuclei were stained with Hoechst 33342. Scale bar = 10 μm. **(F)** Representative Western blots of E-cadherin, occludin, and ZO-1 in HT-29 cells exposed to IL-1β (10 ng/mL) for 24 h in the absence and presence of Scu (10 μM). **(G)** Quantification of E-cadherin, occludin, and ZO-1 protein expression in HT-29 cells after being exposed to IL-1β (10 ng/mL) for 24 h in the absence and presence of Scu (10 μM). Data are expressed as the mean ± SEM. N = 5–6; **, *p* < 0.01 vs. Veh (0.1% DMSO); ^#^, *p* < 0.05, ^##^, *p* < 0.01 vs. IL-1β. One-way ANOVA with Bonferroni’s multiple comparisons tests.

We next investigated whether Scu can affect the epithelial inflammation and epithelial barrier integrity in a cell model. We first examined the cytotoxicity of Scu in cultured HT-29 cells. A 24 h exposure of Scu up to 30 μM had no cytotoxic effect on the HT-29 cell although 100 μM of Scu decreased the HT-29 cell viability by 9.9% ± 1.4% (*p* < 0.01) (Figure 4A). IL-1β is a critical inflammatory mediator in the development of UC (Al-Sadi et al., 2008; Mannino et al., 2019). IL-1β exposure for 12 h dramatically increased the mRNA levels of *IL-6* and *IL-8* to 4.59 ± 0.54 (*p* < 0.01) and 34.69 ± 1.97-fold (*p* < 0.01) of their respective controls (Figures 4B, C). Although the incubation of Scu did not affect the basal expression levels of *IL-6* and *IL-8*, it abolished IL-1β-induced mRNA expression of *IL-6* and decreased that of *IL-8* by 45.8% ± 3.4% (*p* < 0.01) (Figures 4B, C).

IL-1β exposure for 24 h significantly decreased TEER values from 18.76 ± 0.36 Ω⋅cm^2^ to 13.26 ± 0.78 Ω⋅cm^2^ (*P* < 0.01) in cultured HT-29 cell monolayer (Figure 4D). Although Scu (10 μM) treatment alone did not alter the TEER value, it significantly increased TEER values to 16.45 ± 1.15 Ω⋅cm^2^ (*p* < 0.05) in IL-1β-treated HT-29 cell monolayer (Figure 4D). HT-29 monolayer cells showed considerable staining of E-cadherin, occludin, and ZO-1 proteins in the cell membrane between cells, consistent with their function to maintain barrier integrity (Figure 4E). IL-1β exposure decreased the immunofluorescence signals of E-cadherin, occludin, and ZO-1 (Figure 4E). While Scu (10 μM) treatment for 24 h did not alter the expression levels and distribution patterns of these tight junction proteins in vehicle (0.1% DMSO)-treated HT-29 monolayer, it increased the expression levels of E-cadherin, occludin, and ZO-1 in IL-1β-treated HT-29 cell monolayer from 0.66 ± 0.06 to 1.19 ± 0.1 (*p* < 0.01), 0.74 ± 0.04 to 0.99 ± 0.06 (*p* < 0.01), and 0.66 ± 0.05 to 0.95 ± 0.08-fold (*p* < 0.01) of their respective controls (Figures 4E–G).

### Scu suppressed NF-κB signaling in HT-29 cells to decrease epithelial inflammation and maintain epithelial barrier integrity

**FIGURE 5:**
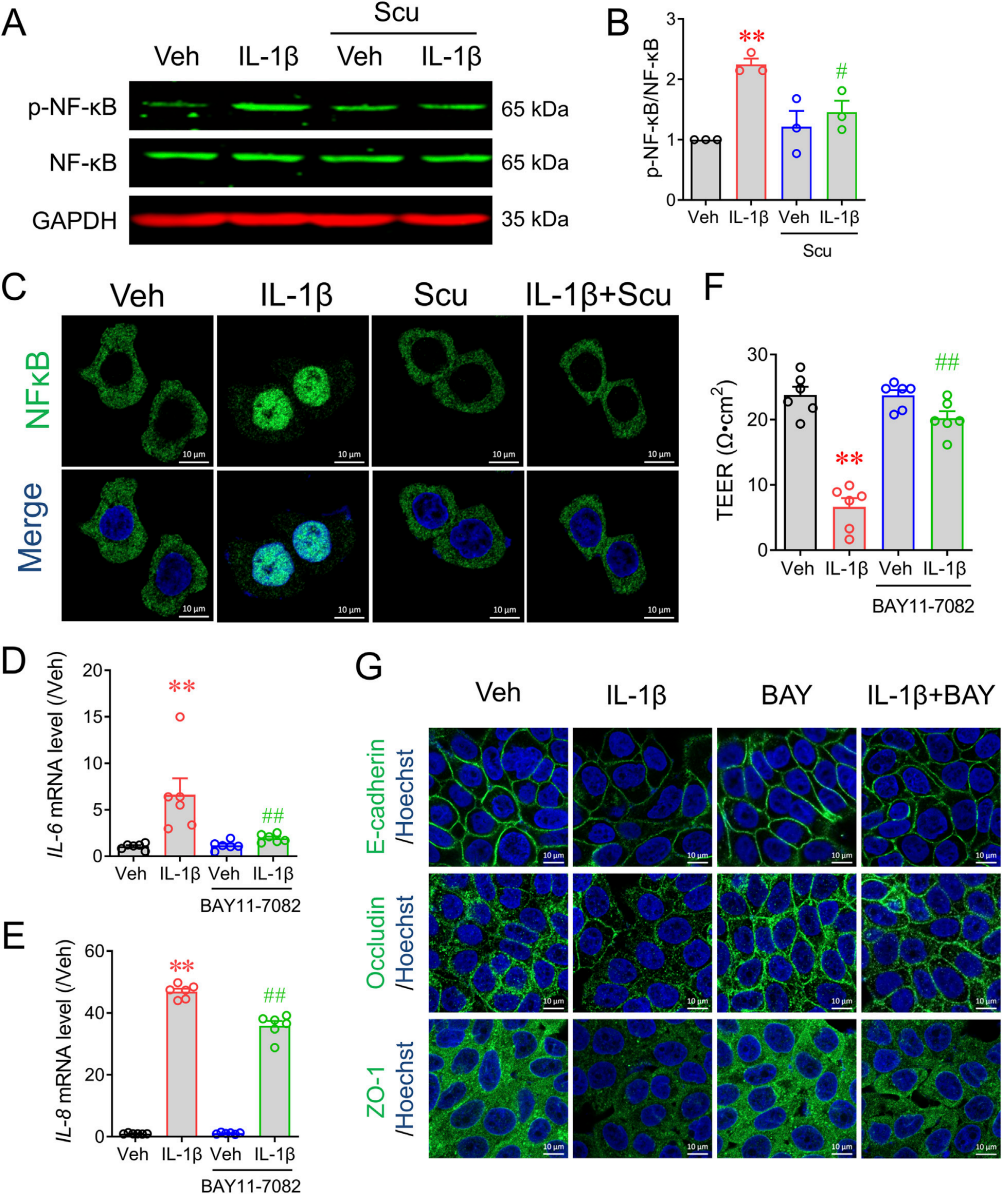
Scu inhibited IL-1β-induced phosphorylation of NF-κB and its nuclear translocation in HT-29 cells. **(A)** Representative Western blots and **(B)** quantification of phosphorylated NF-κB in HT-29 cells. HT-29 cells were exposed to IL-1β (10 ng/mL) in the absence and presence of Scu (10 μM). **(C)** Representative immunofluorescence images of stained with NF-κB in HT-29 cells. Cells were treated with IL-1β (10 ng/mL) for 30 min in the absence and presence of Scu (10 μM). Nuclei were stained with Hoechst 33342. Scale bar = 10 μm. **(D, E)** Quantitative RT-PCR analysis of mRNA levels of *IL-6* **(D)** and *IL-8* **(E)** in HT-29 cells treated with IL-1β (10 ng/mL) for 12 h in the absence and presence of BAY11-7082 (3 μM). **(F)** Changes in the TEER of HT-29 monolayer treated with IL-1β (10 ng/mL) for 24 h in the absence and presence of BAY11-7082 (3 μM). **(G)** Representative immunofluorescence images of E-cadherin, occludin, and ZO-1 in HT-29 cells exposed to IL-1β (10 ng/mL) for 24 h in the absence and presence of BAY11-7085 (3 μM). Nuclei were stained with Hoechst 33342. Scale bar = 10 μm. N = 3–6. **, *p* < 0.01 vs. Veh; ^#^, *p* < 0.05, ^##^, *p* < 0.01 vs. IL-1β. One-way ANOVA with Bonferroni’s multiple comparisons tests.

We next explored how Scu exerted its anti-inflammatory on epithelial HT-29 cells. The activation of the nuclear transcription factor NF-κB mediates signaling pathways for a variety of inflammatory mediators, including IL-1β (Lee et al., 2024). Western blot analysis demonstrated that IL-1β exposure rapidly increased NF-κB phosphorylation (Figure 5A) and nuclear translocation (Figure 5C). Treatment with Scu (10 μM) for 30 min significantly suppressed IL-1β-induced NF-κB phosphorylation by 63.4% ± 10.8% (*p* < 0.01) (Figure 5B) and abrogated the IL-1β-induced NF-κB nuclear translocation (Figure 5C). To validate the promotion of NF-κB activation on epithelial inflammation and barrier integrity, we examined the effects of NF-κB inhibition on IL-1β-induced *IL-6* and *IL-8* expression and barrier disruption. An NF-κB inhibitor, BAY-11–7082 (3 μM) suppressed IL-1β upregulated mRNA expression of *IL-6* and *IL-8* by 84.1% ± 3.2% (*p* < 0.01) and 24.1% ± 3.3% (*p* < 0.01), respectively (Figures 5D,E). BAY-11–7082 also reversed the value of TEER in IL-1β-treated HT-29 cells from 6.60 ± 1.35 Ω·cm^2^ to 20.24 ± 1.07 Ω·cm^2^ (*p* < 0.01) (Figure 5F) and IL-1β downregulated protein expression of E-cadherin, occludin, and ZO-1 (Figure 5G).

## Discussion

Scu is one of the main active flavonoids found in multiple traditional Chinese medicines. Scu has been demonstrated to alleviate chronic obstructive pulmonary diseases (Liu et al., 2023), cardiac hypertrophy (Shi et al., 2022), pulmonary fibrosis (Miao et al., 2020), liver inflammation (Lan et al., 2023), and osteoarthritis (Ye et al., 2024). We have also demonstrated that Scu alleviates DNFB and carvacrol-induced pruritus and dermatitis (Wang et al., 2022). In the current study, we found that Scu effectively ameliorated DSS-induced weight loss, colon atrophy, histopathological damage, and colonic inflammation in mice, therefore extending its potential utility to treat UC. It should be noted that at a dose of 20 mg/kg, Scu displayed better protection on DSS-induced UC severity, inflammation, and epithelial barrier disruption than the positive drug, 5-ASA, suggesting its superior efficacy in the treatment of UC.

Although UC is a chronic disease manifested by dysregulated immune response, gut homeostasis coordinates the interplay between immune cells and bacteria. The disruption of the gut barrier with subsequent loss of mucosal homeostasis is a critical step to trigger intestinal inflammation (Van der Post et al., 2019). In the current study, we also demonstrated that Scu effectively suppressed the epithelial barrier damage in DSS-treated mice as reflected by the restoration of DSS-disrupted expression of tight junction proteins, as well as in the IL-1β-treated epithelial HT-29 cell monolayer. Treatment of Scu completely reversed the IL-1β-induced decrease in the TEER value and the downregulated expression levels of tight junction proteins such as E-cadherin, occludin, and ZO-1. These data demonstrated that in both *in vivo* and *in vitro* models, Scu was capable of preserving the integrity of the epithelial barrier. Upon pathogen/inflammatory mediator stimulation, intestinal epithelial cells also release a variety of proinflammatory mediators (Mannino et al., 2019). These proinflammatory mediators further aggravate the infiltration of immune cells, contributing to the progression of intestinal inflammation. We demonstrated that in HT-29 cultures, Scu completely suppressed the IL-1β-upregulated *IL-6* expression although it was less effective on the IL-1β-upregulated *IL-8* expression. Although Scu has been demonstrated to suppress LPS-induced inflammation in RAW264.7 macrophages, considering its relatively low absorption and low level in the blood (Zhang et al., 2016), it is feasible to prospect that Scu likely exerts its effect in the gut, possibly by preserving the epithelial barrier integrity and inhibiting proinflammatory response of the epithelial cells. Such combinatorial effects on barrier disruption and anti-inflammation may explain the superior therapeutic efficacy of Scu to 5-ASA on UC.

During the progression, IL-1β is a critical inflammatory mediator that causes intestinal inflammation and disruption of epithelial integrity (Al-Sadi et al., 2008). As one of the major mediators, NF-κB is critical to inflammation in epithelial cells (Laurindo et al., 2023). Consistent with the previous results, we demonstrated that IL-1β exposure increased NF-κB phosphorylation and nuclear translocation, while the suppression of NF-κB phosphorylation suppressed the IL-1β-upregulated *IL-6* and *IL-8* mRNA expression. The NF-κB inhibitor, BAY11-7082, also reversed the IL-1β-induced decreases in TEER values and the expression of E-cadherin, occludin, and ZO-1. Interestingly, with the Scu treatment, IL-1β-induced NF-κB phosphorylation and nuclear translocation were markedly suppressed. Considered together these data demonstrate that Scu exerts its protection on epithelial barrier integrity and anti-inflammatory effect through suppression of the NF-κB signaling pathway. However, the molecular target remains to be explored. We previously demonstrated that Scu is a selective inhibitor of TRPV3 and Scu alleviates DNFB and carvacrol-induced pruritus and dermatitis through specific suppression of TRPV3 (Wang et al., 2022). A recent study has reported the abundant expression of TRPV3 in the colon epithelial cells (Ueda et al., 2009). It remains to be established that whether Scu’s effect is, at least in part, due to its inhibition of TRPV3 activity.

## Conclusion

**FIGURE 6:**
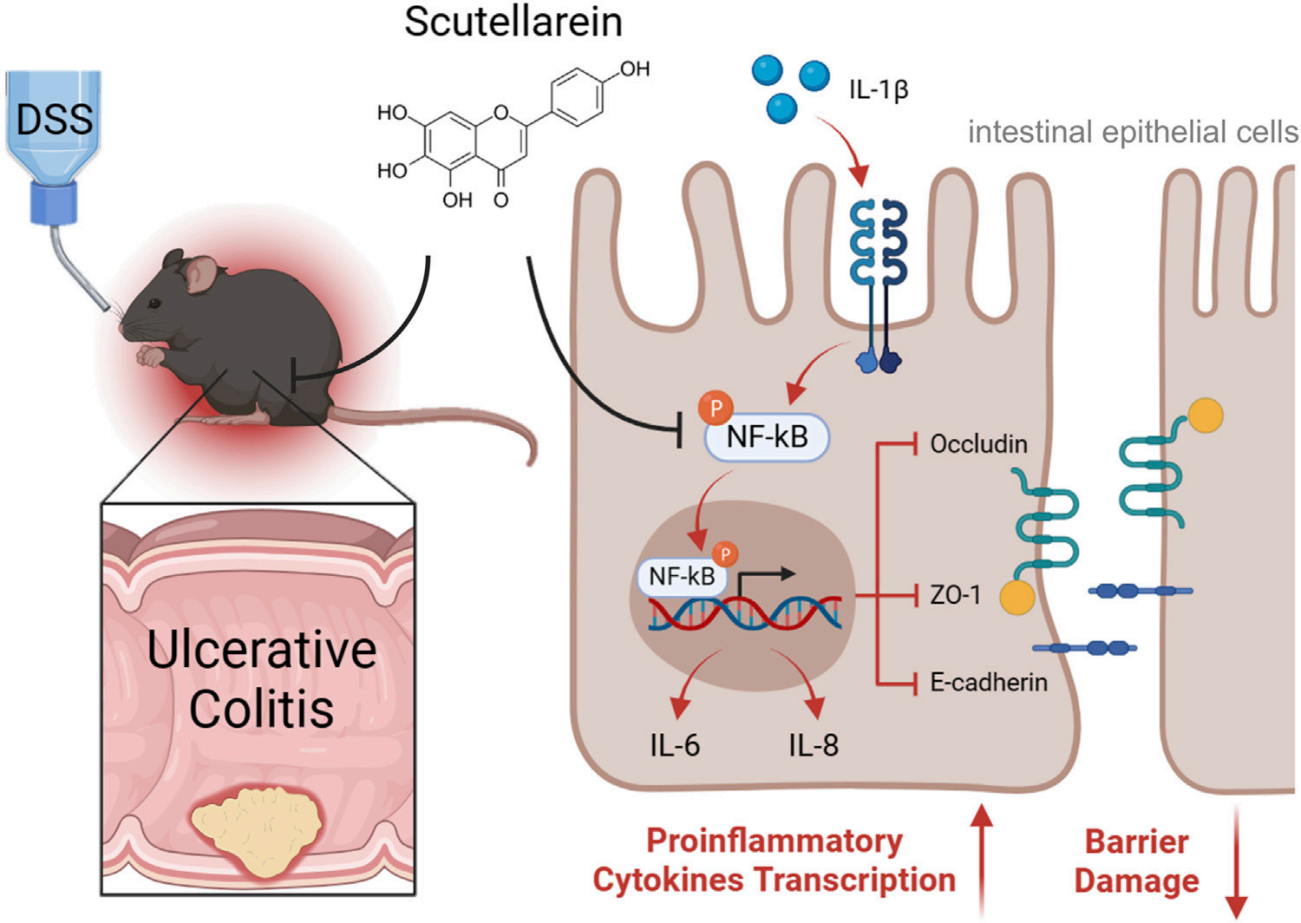
Schematic depiction of Scu amelioration of DSS-induced ulcerative colitis in mice. Scu alleviates DSS-induced UC through its suppression of proinflammatory mediators and preservation of epithelial barrier integrity by inhibiting NF-κB signaling in intestinal epithelial cells.

The current study expands on the potential utility of Scu in treating UC. Scu exerts its protective effect on UC through its anti-inflammatory effect on intestinal epithelial cells and its ability to preserve epithelial barrier integrity (Figure 6). Scu has the potential to be a novel therapeutic agent for the treatment of UC.

## Data Availability

The raw data supporting the conclusions of this article will be made available by the authors, without undue reservation.

## Abbreviations

5-ASA, 5-aminosalicylic acid; CMC-Na, sodium carboxymethyl cellulose; CXCL1, C-X-C motif chemokine ligand 1; DAI, disease activity index; DMSO, dimethyl sulfoxide; DNFB, 2,4-dinitrofluorobenzene; DSS, dextran sodium sulfate; IL, interleukin; IBD, inflammatory bowel disease; MTT, 3-(4,5)-dimethylthiahiazo (-z-y1)-3,5-di-phenytetrazoliumromide; MPO, myeloperoxidase; NF-κB, nuclear factor kappa B; Scu, scutellarein; TNF-α, tumor necrosis factor-α; TJ, tight junction; TEER, transepithelial electrical resistance; UC, ulcerative colitis; ZO-1, zona occludens 1.
