# Supplementary material for: Scutellarein ameliorates dextran sulfate sodium-induced ulcerative colitis by inhibiting colonic epithelial cell proinflammation and barrier disruption
Source: Front Pharmacol. 2024 Oct 21;15:1479441. doi: 10.3389/fphar.2024.1479441 (PMC11536309; doi:10.3389/fphar.2024.1479441)
Supplement: Supplementary file 1 [file DataSheet1.DOC]

**Unedited blot and gel images:**

**Scutellarein Ameliorates Dextran Sulfate Sodium-Induced Ulcerative Colitis by inhibiting Colonic Epithelial Cell Proinflammation and Barrier Disruption**

The original full blots for E-cadherin in **Figure 3B**. Red boxes indicate the bands used in the figure.

**1**


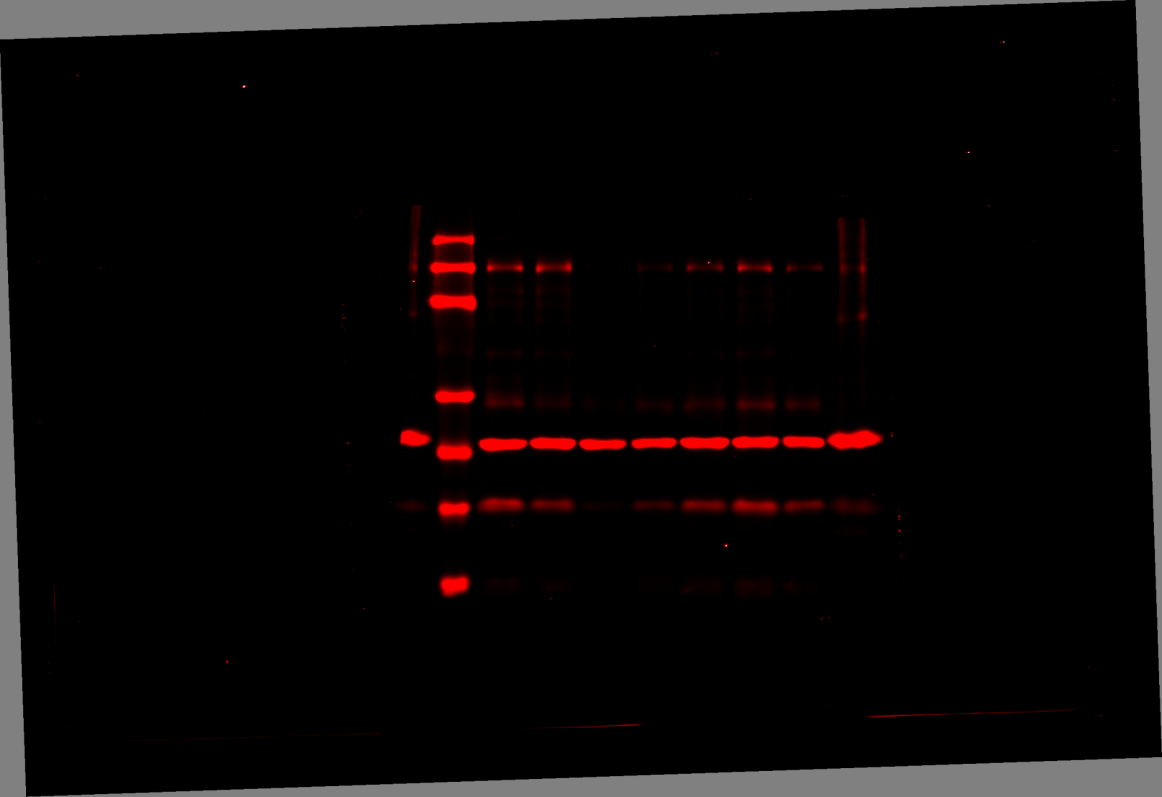


kDa

180

130

100

70

55

40

35

25

β-actin

**
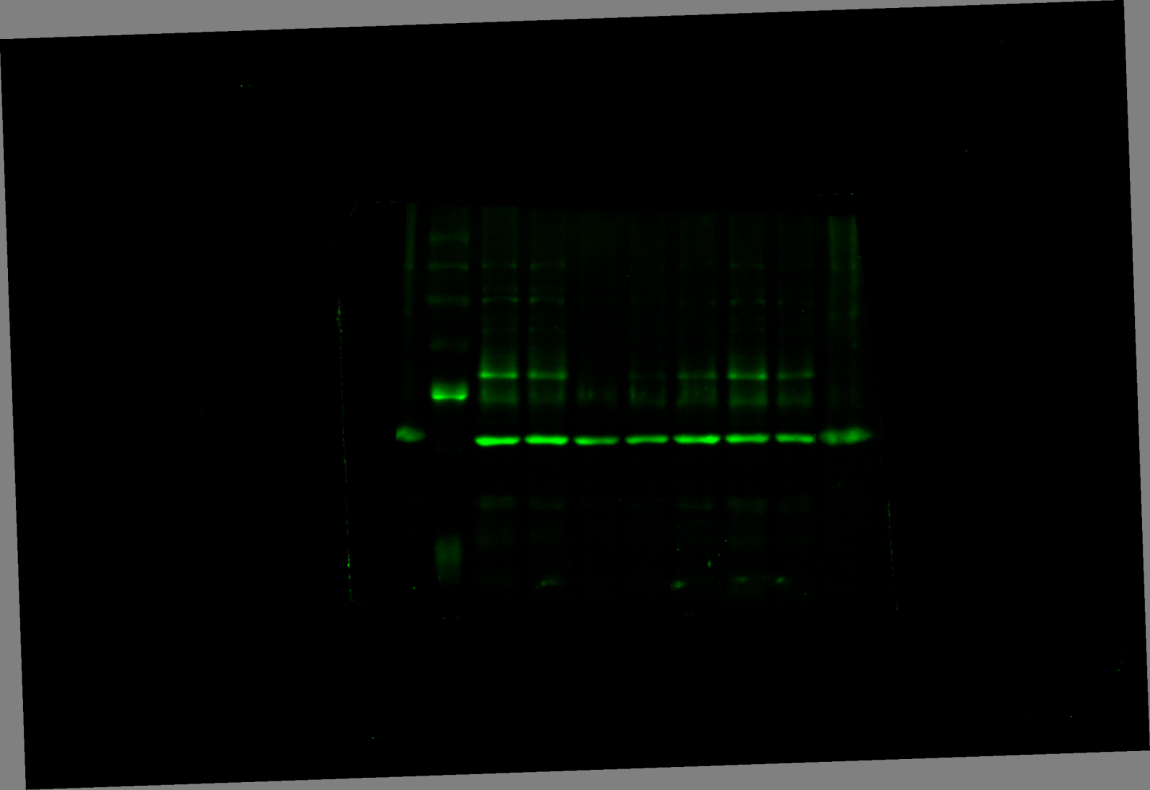

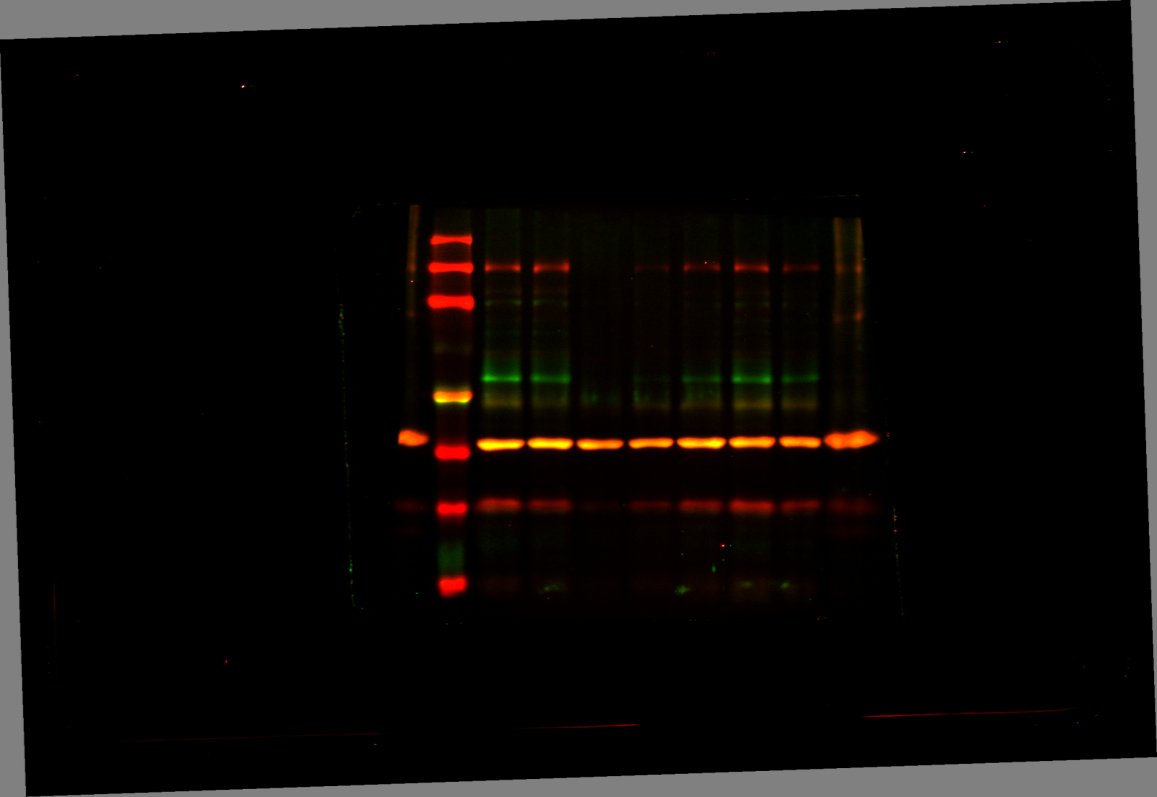
**

E-cadherin

Veh

DSS

S20

S5+D

S10+D

S20+D

ASA+D

β-actin

**2** **3** **4**


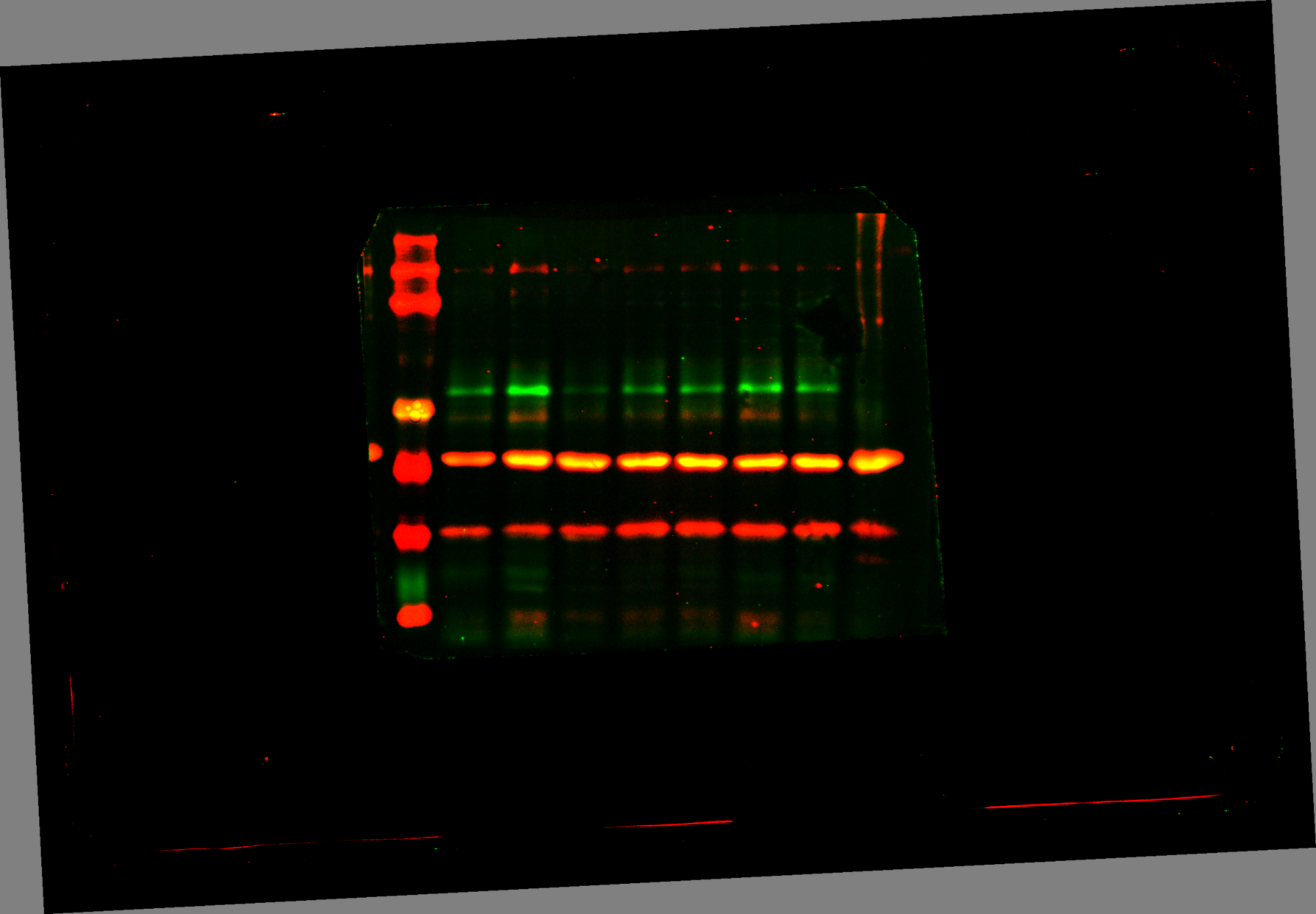


E-cadherin

β-actin


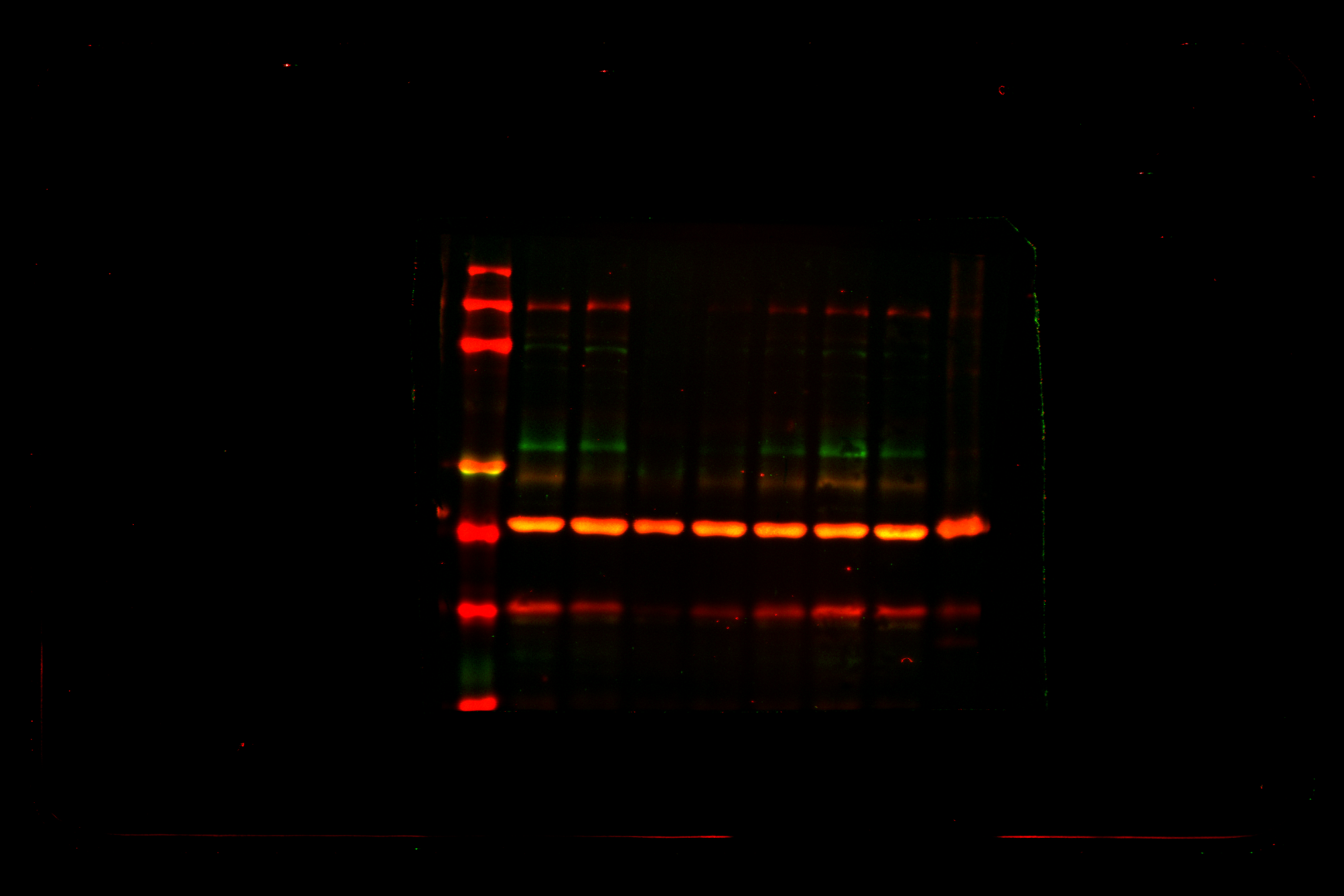


E-cadherin

β-actin


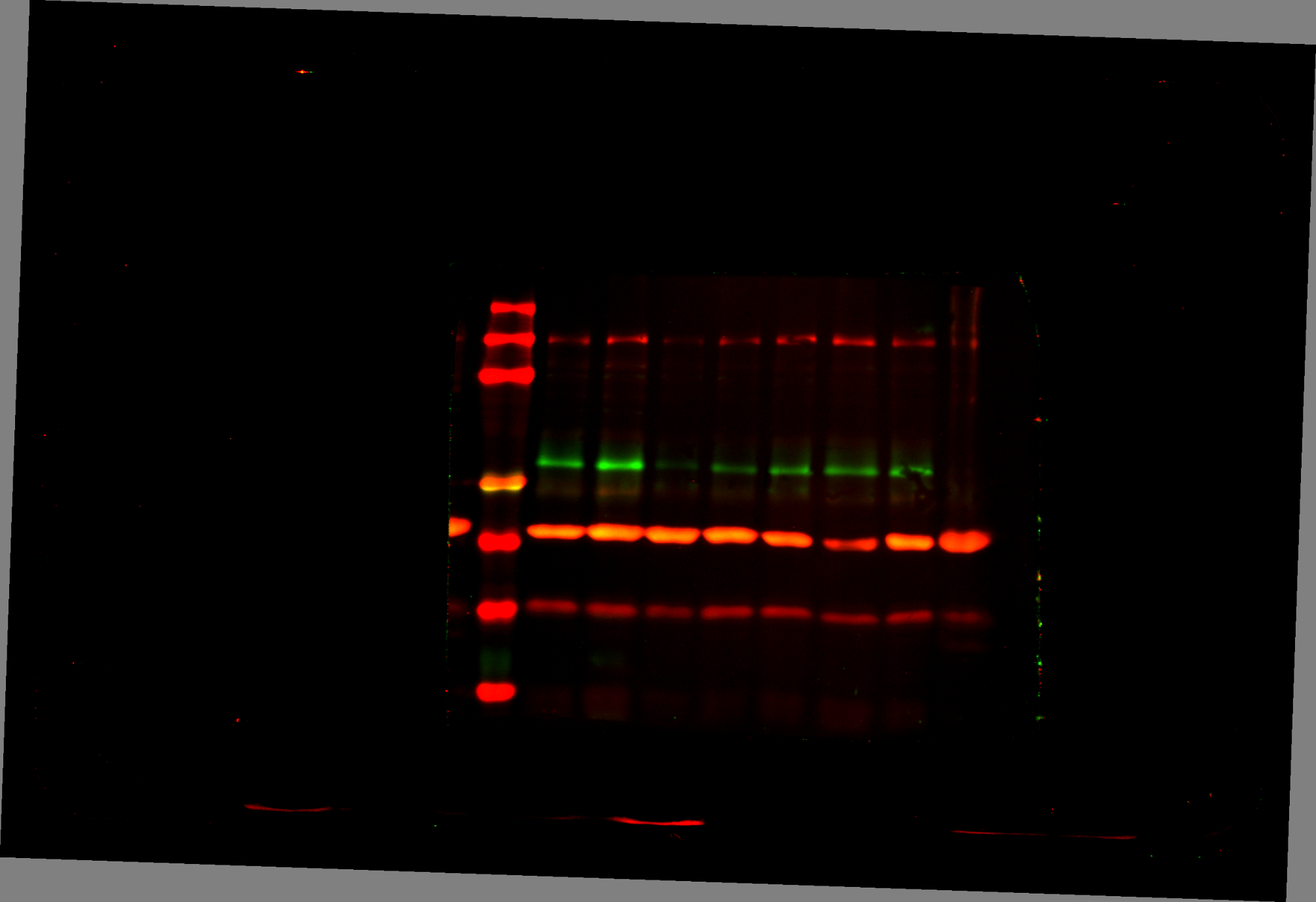


E-cadherin

β-actin

**5** **6**


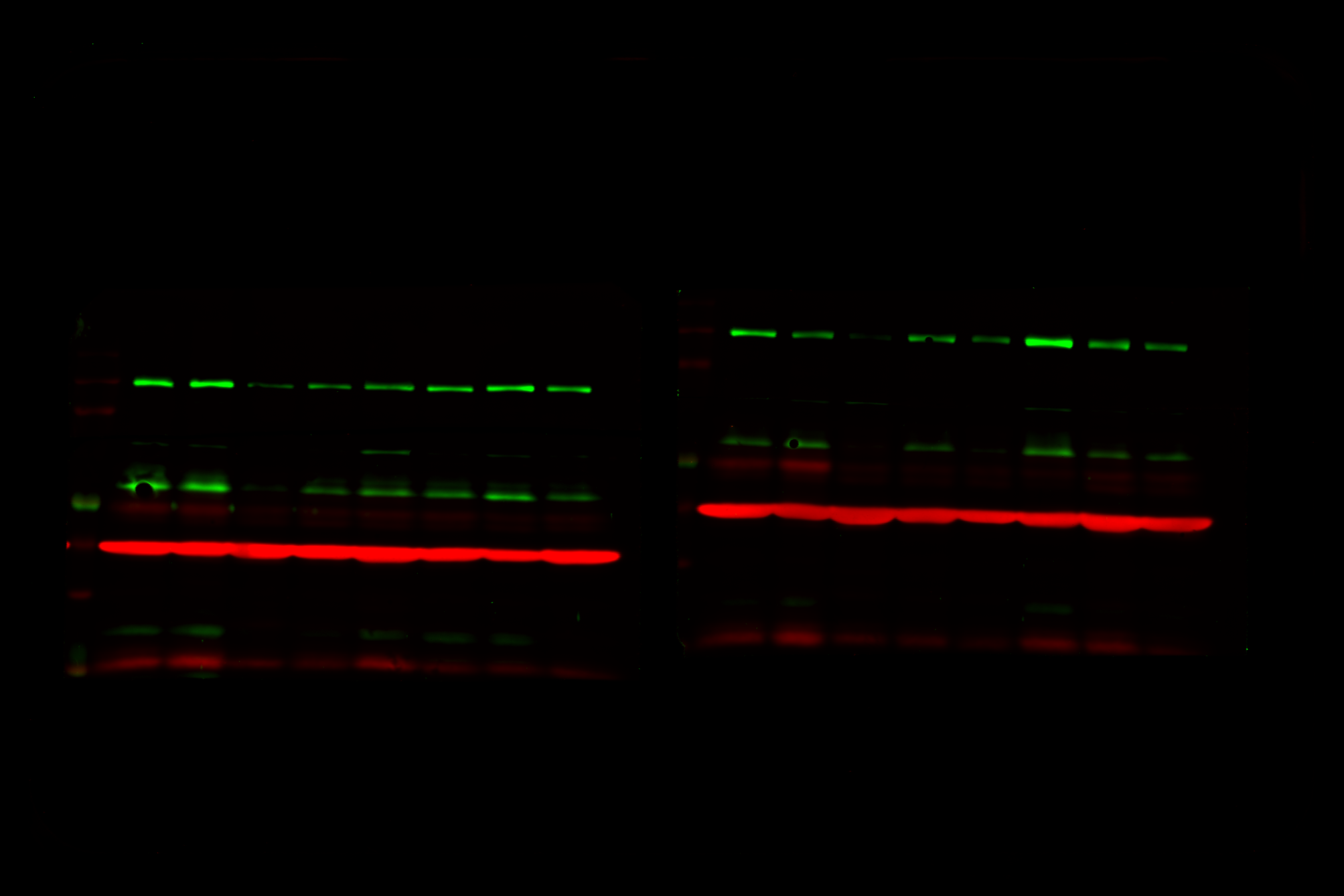


E-cadherin

β-actin


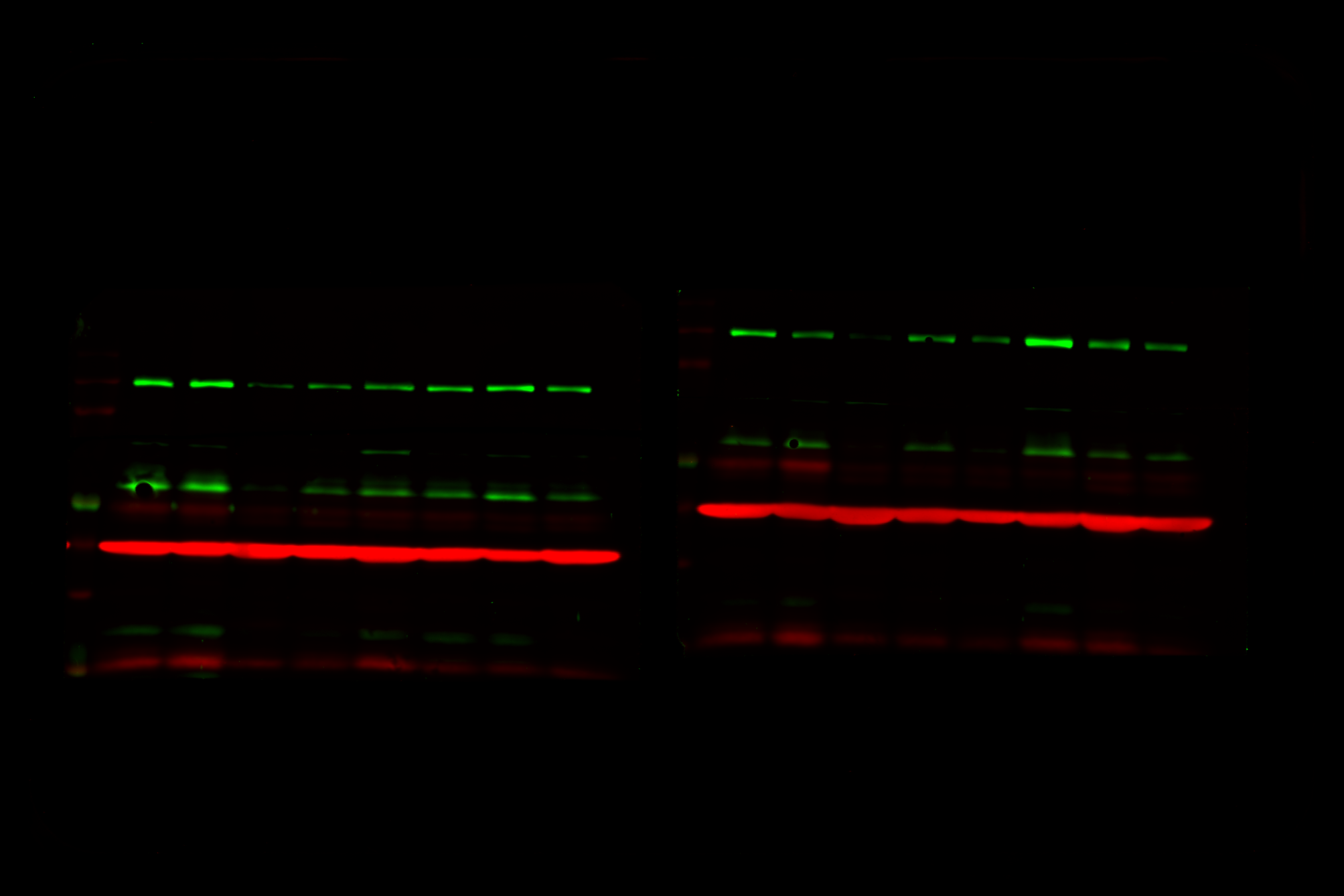


E-cadherin

β-actin

The original full blots for Occludin in **Figure 3B**. Red boxes indicate the bands used in the figure.

**1**


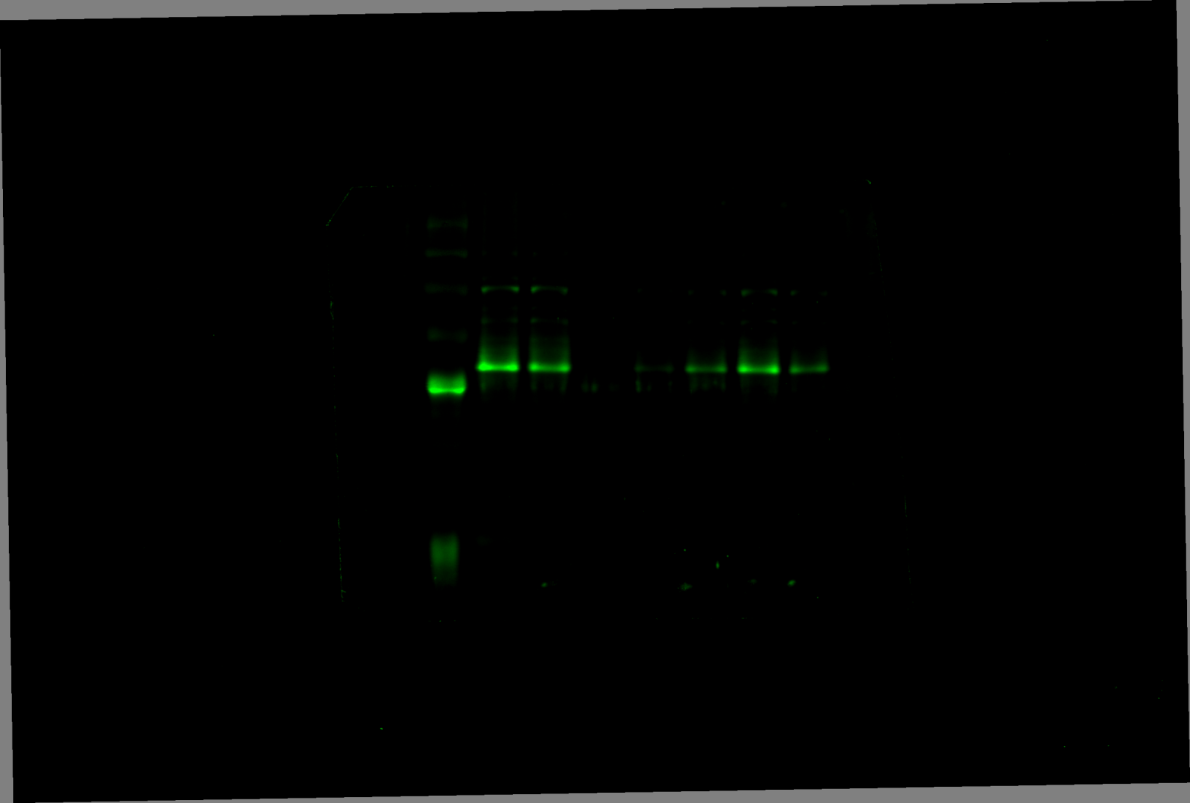

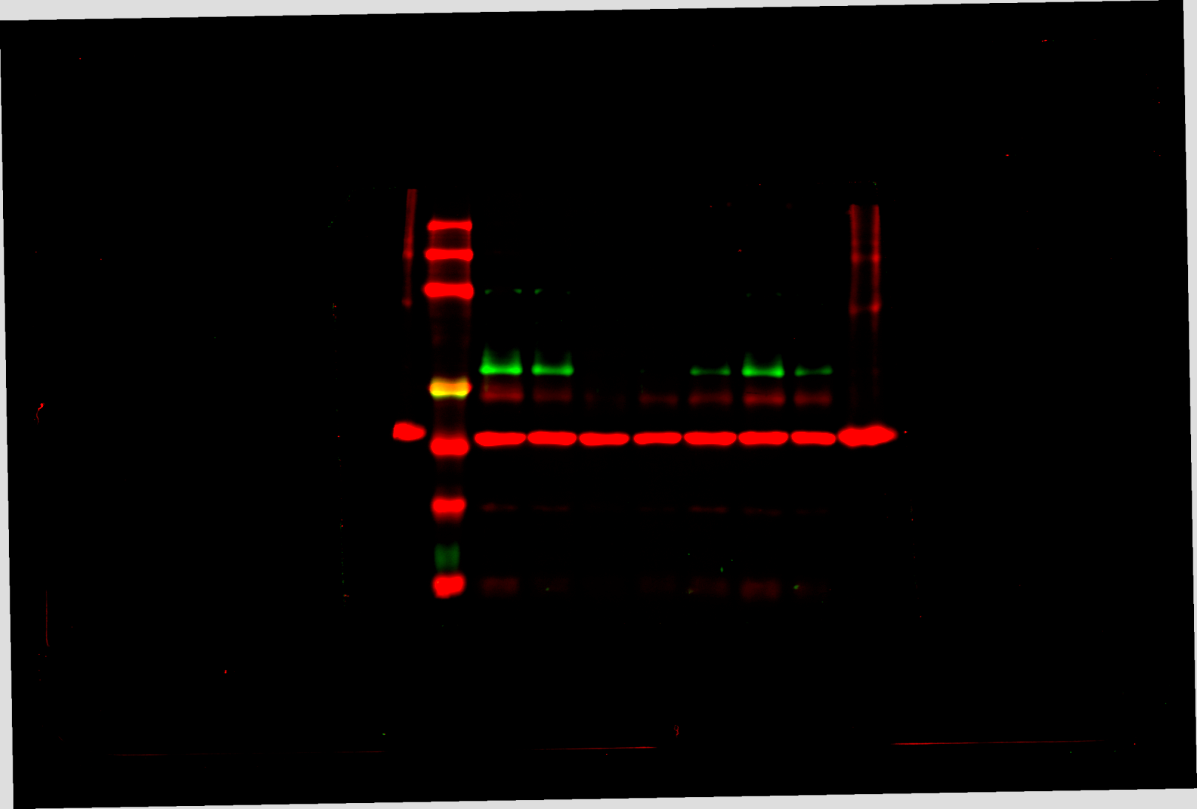

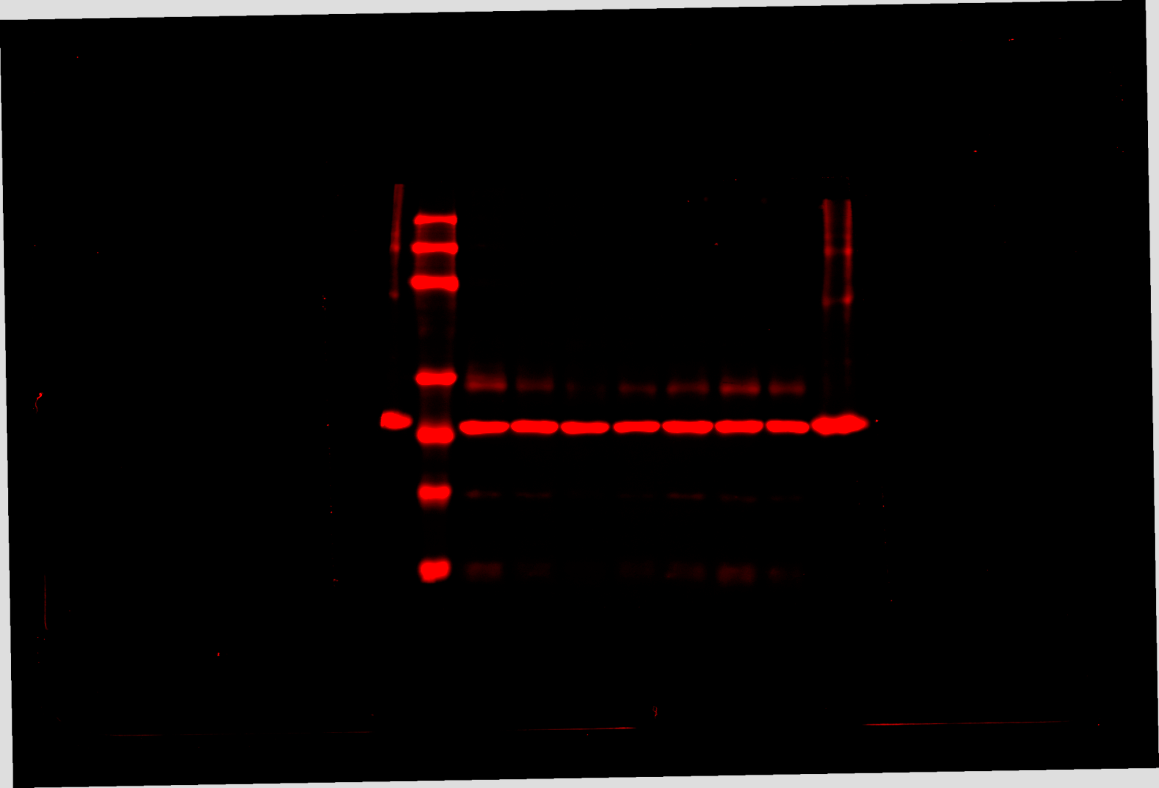


kDa

180

130

100

70

55

40

35

25

β-actin

Veh

DSS

S20

S5+D

S10+D

S20+D

ASA+D

Occludin

**
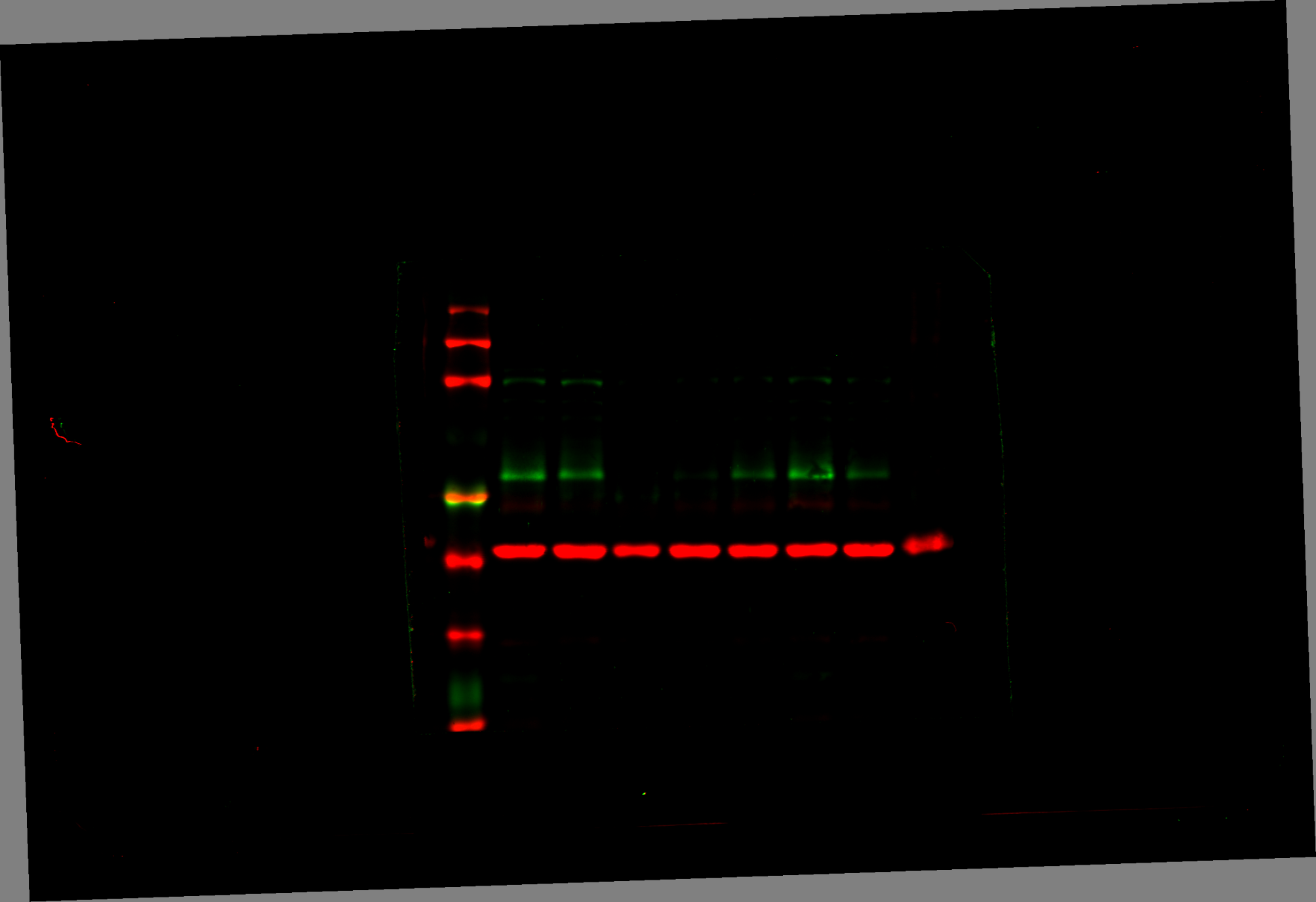

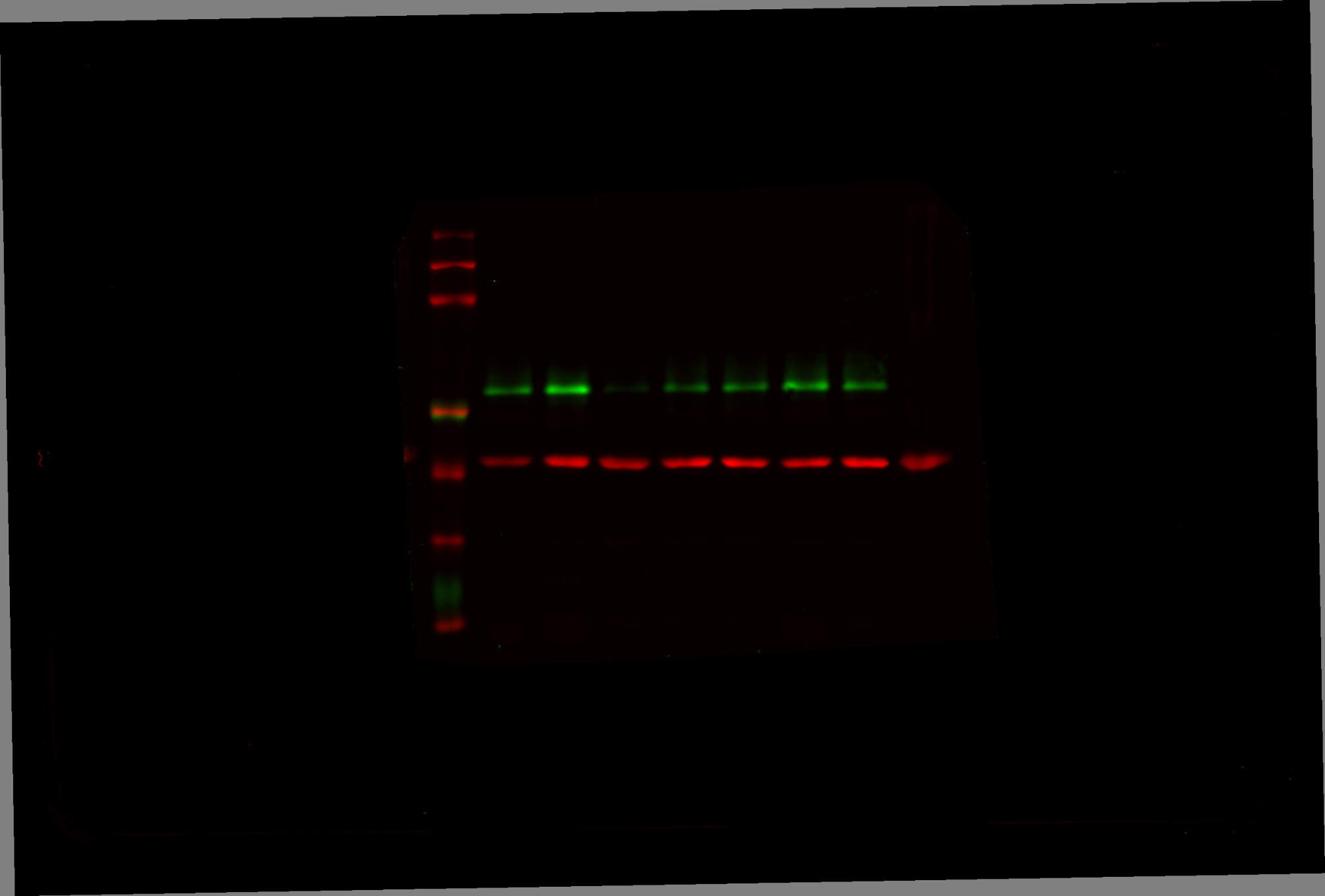

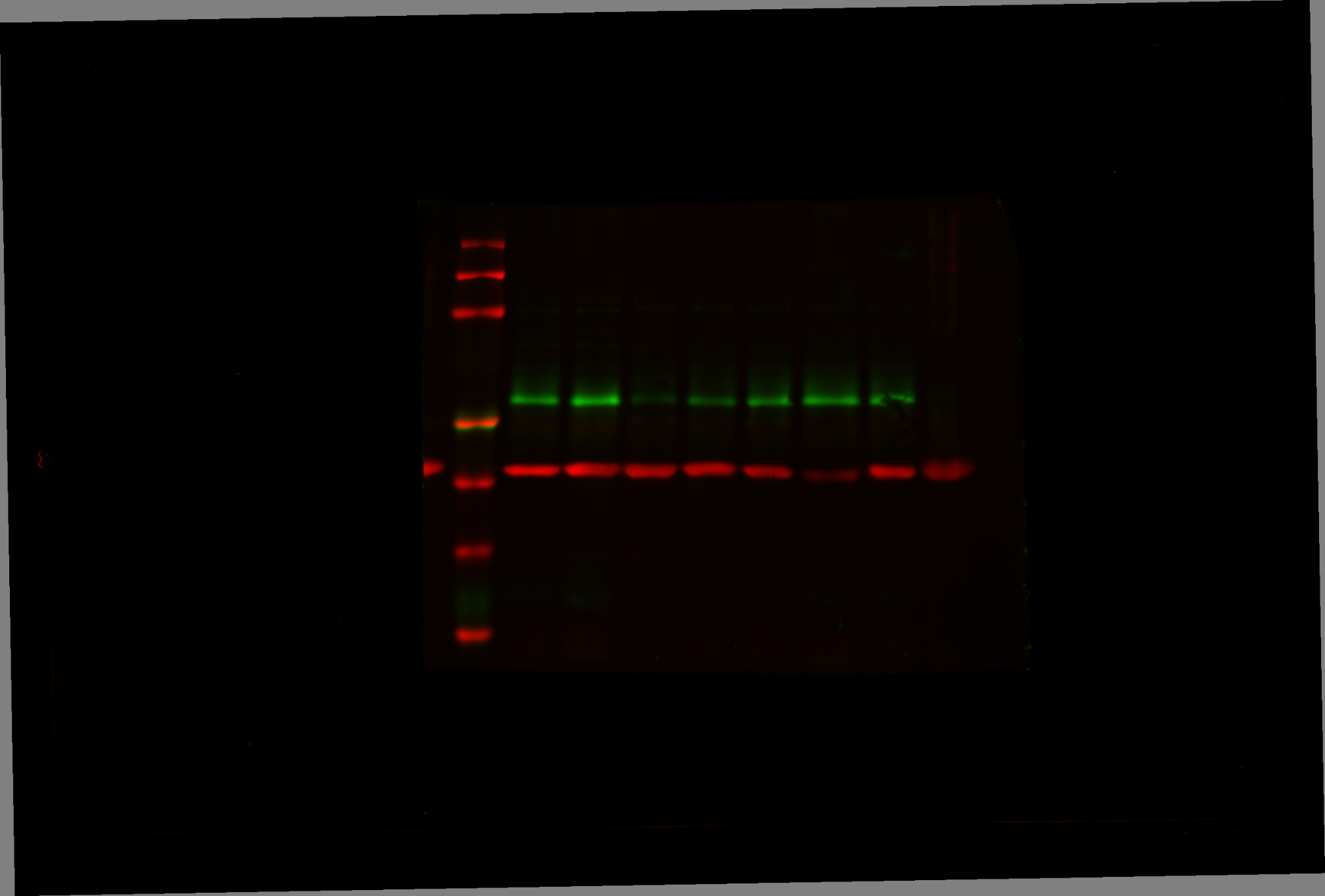
2 3 4**

Occludin

Occludin

Occludin

β-actin

β-actin

β-actin

**5 6**

**
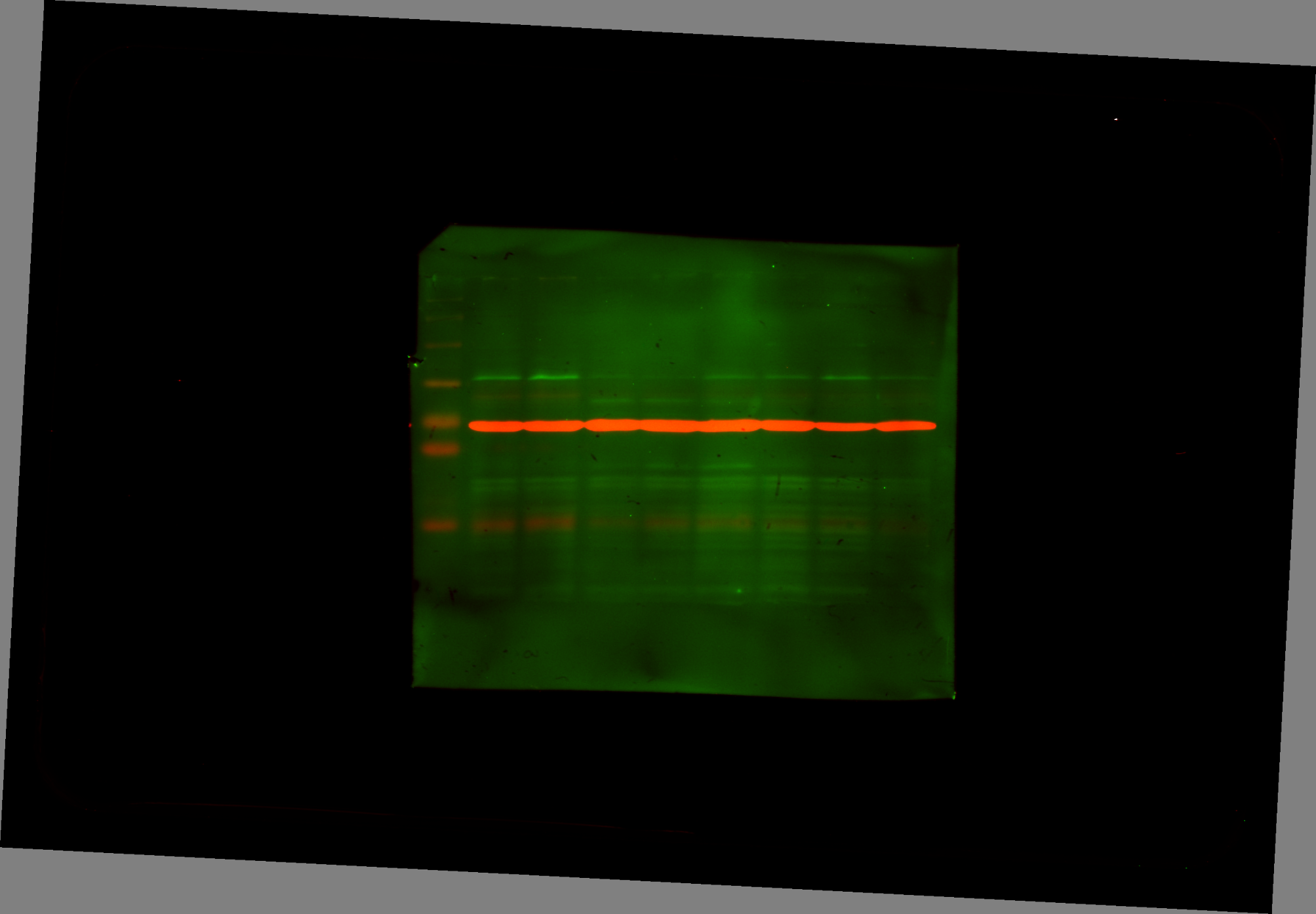

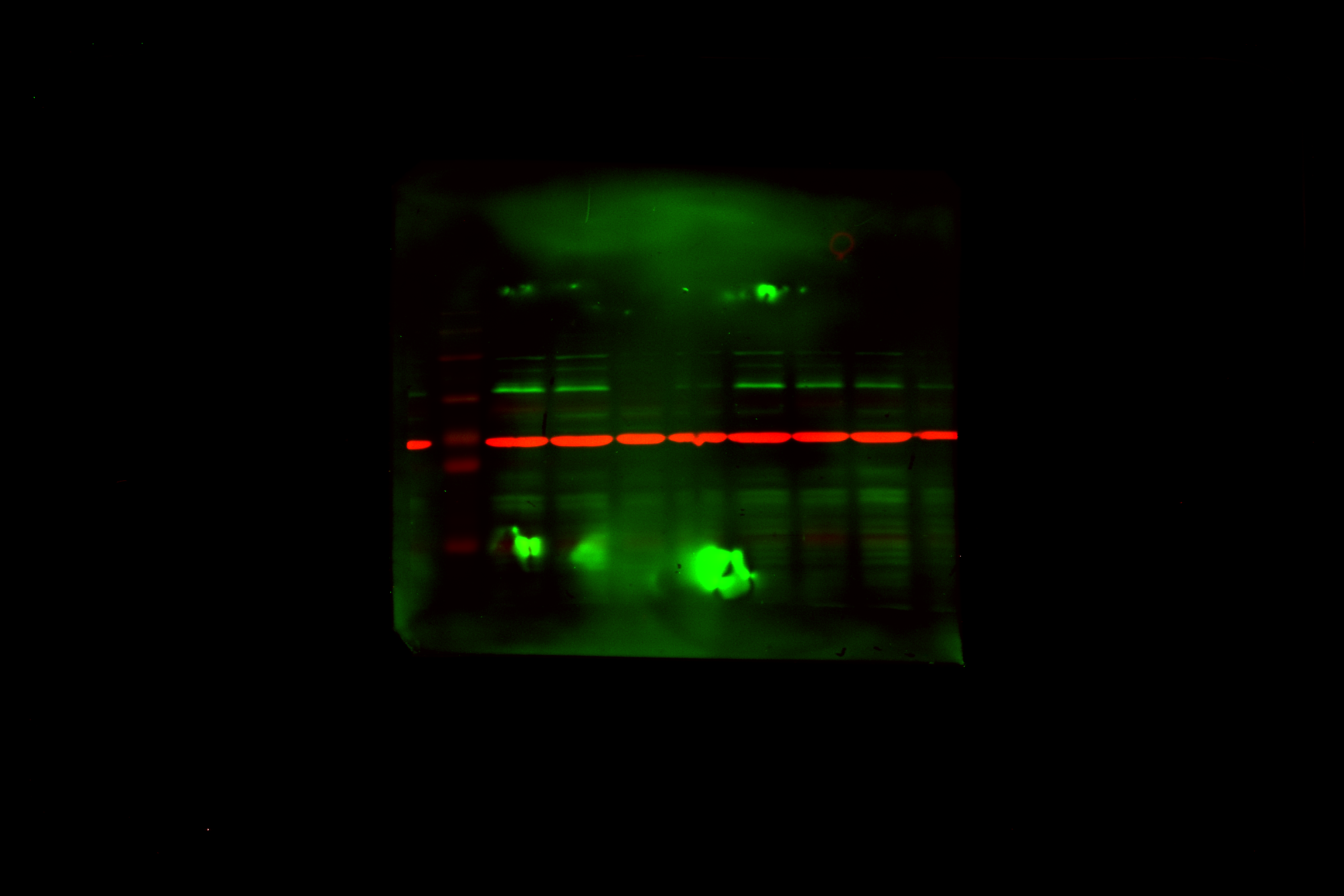
**

Occludin

Occludin

β-actin

β-actin

The original full blots for ZO-1 in **Figure 3B**. Red boxes indicate the bands used in the figure.

**1**

β-actin


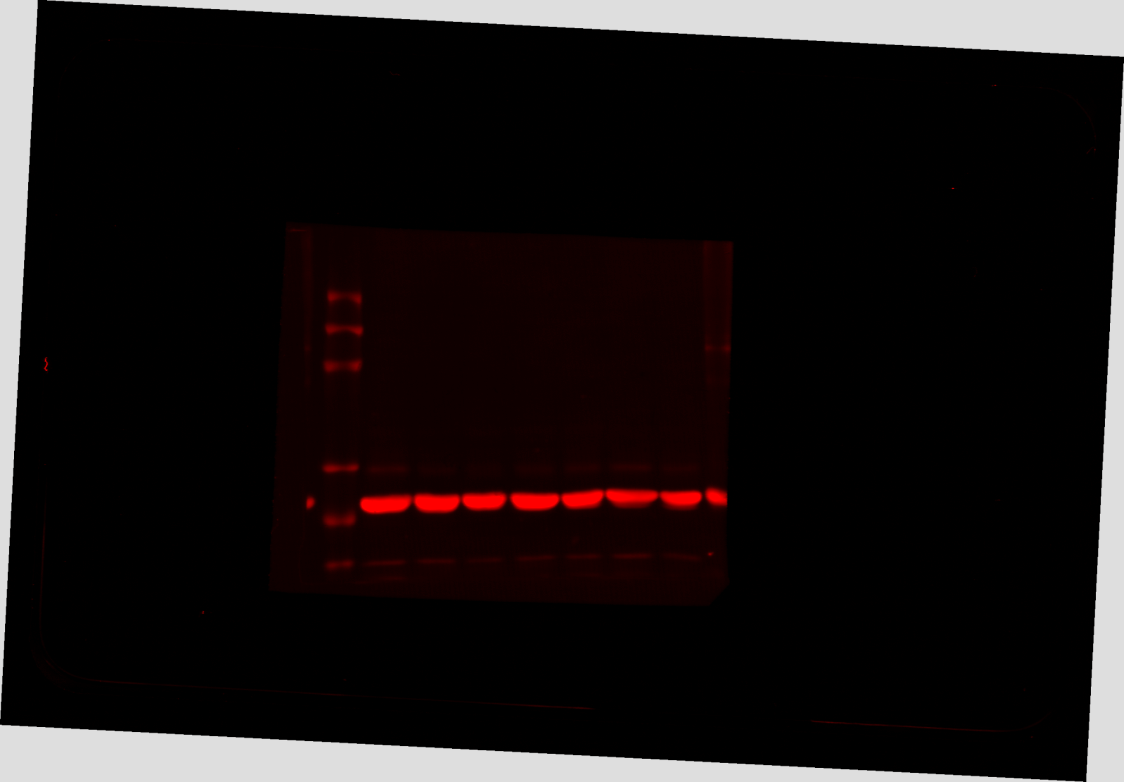


β-actin


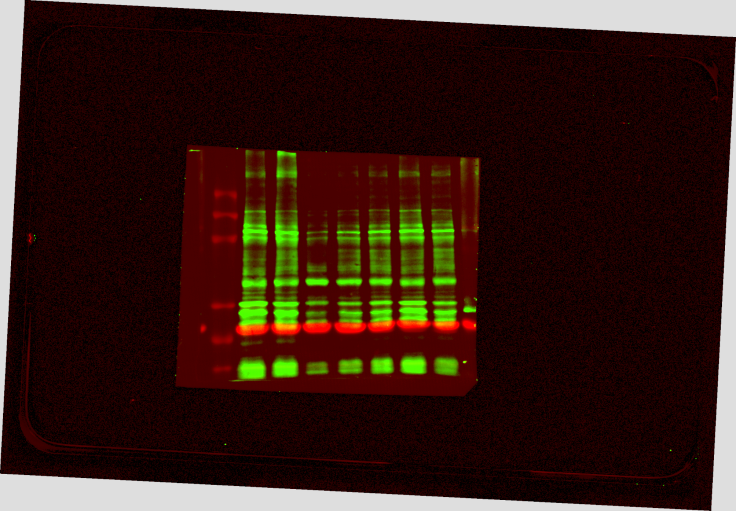

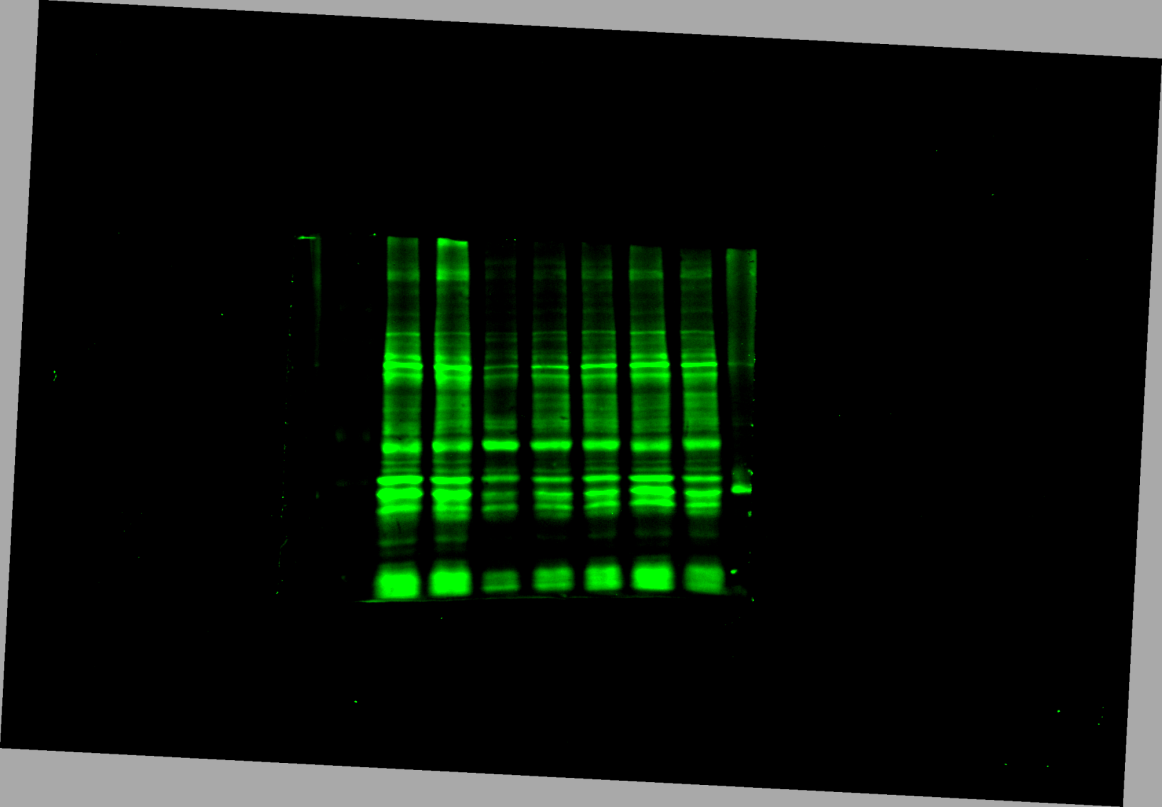


Veh

DSS

S20

S5+D

S10+D

S20+D

ASA+D

ZO-1

kDa

200

130

95

70

50

40

35


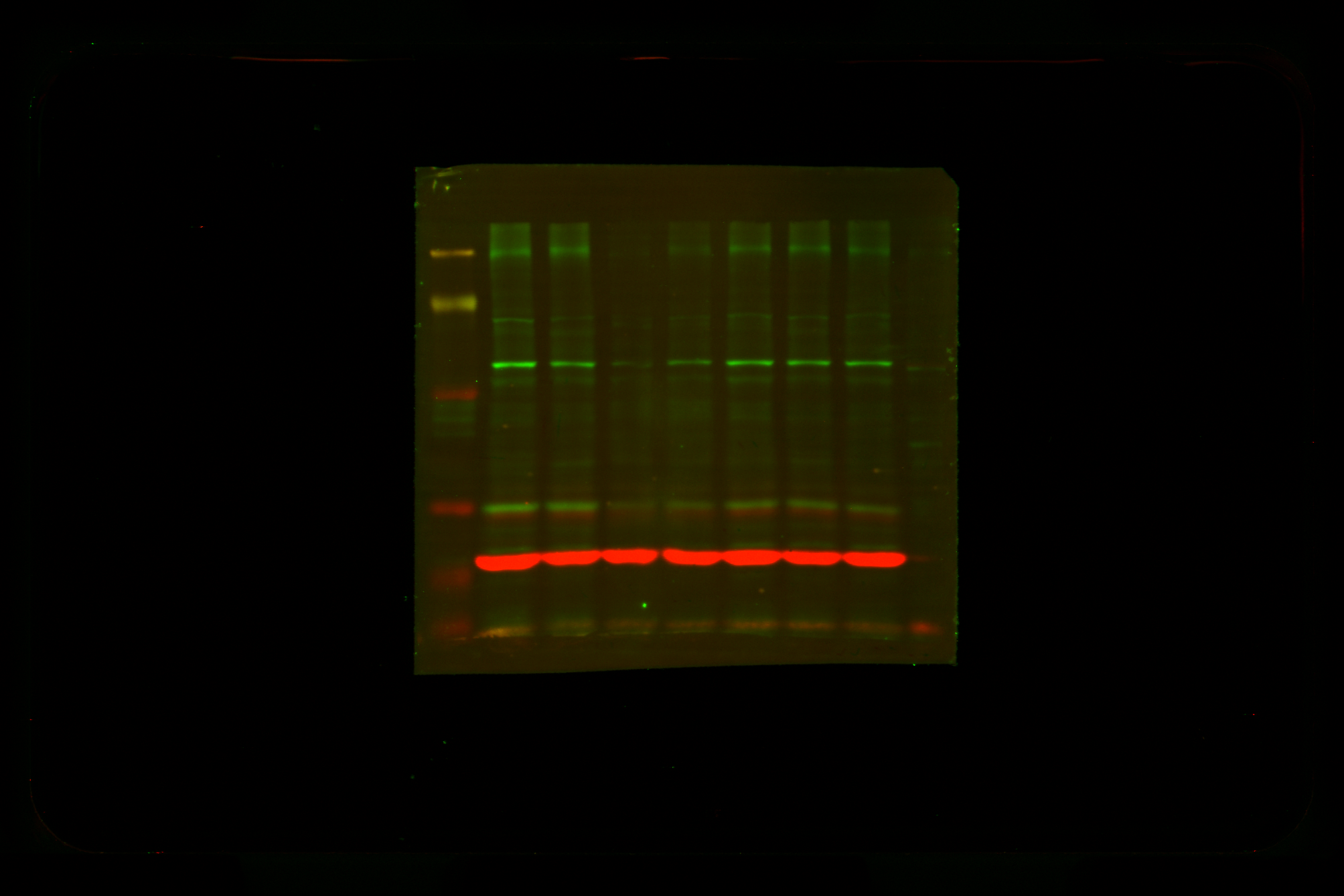

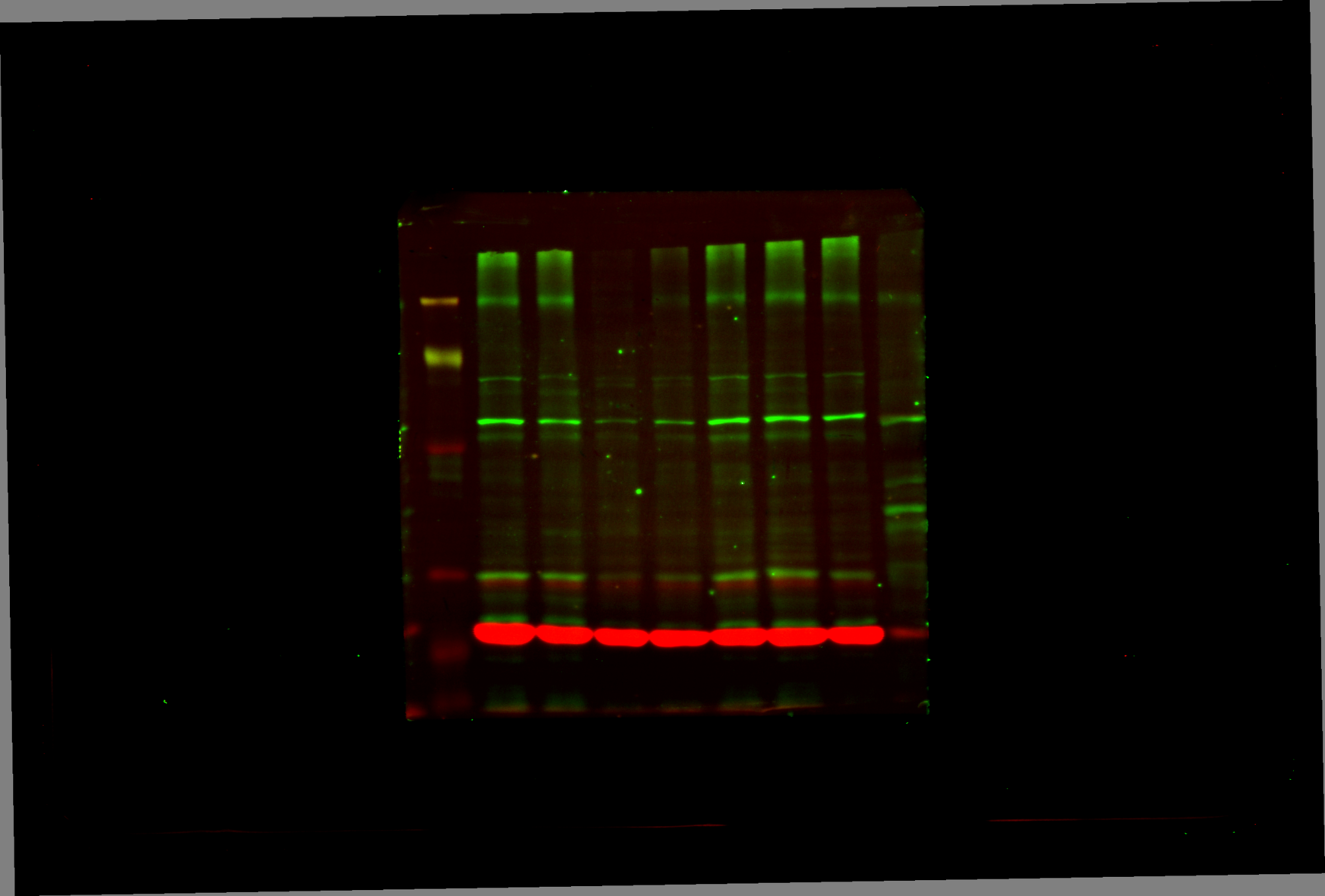

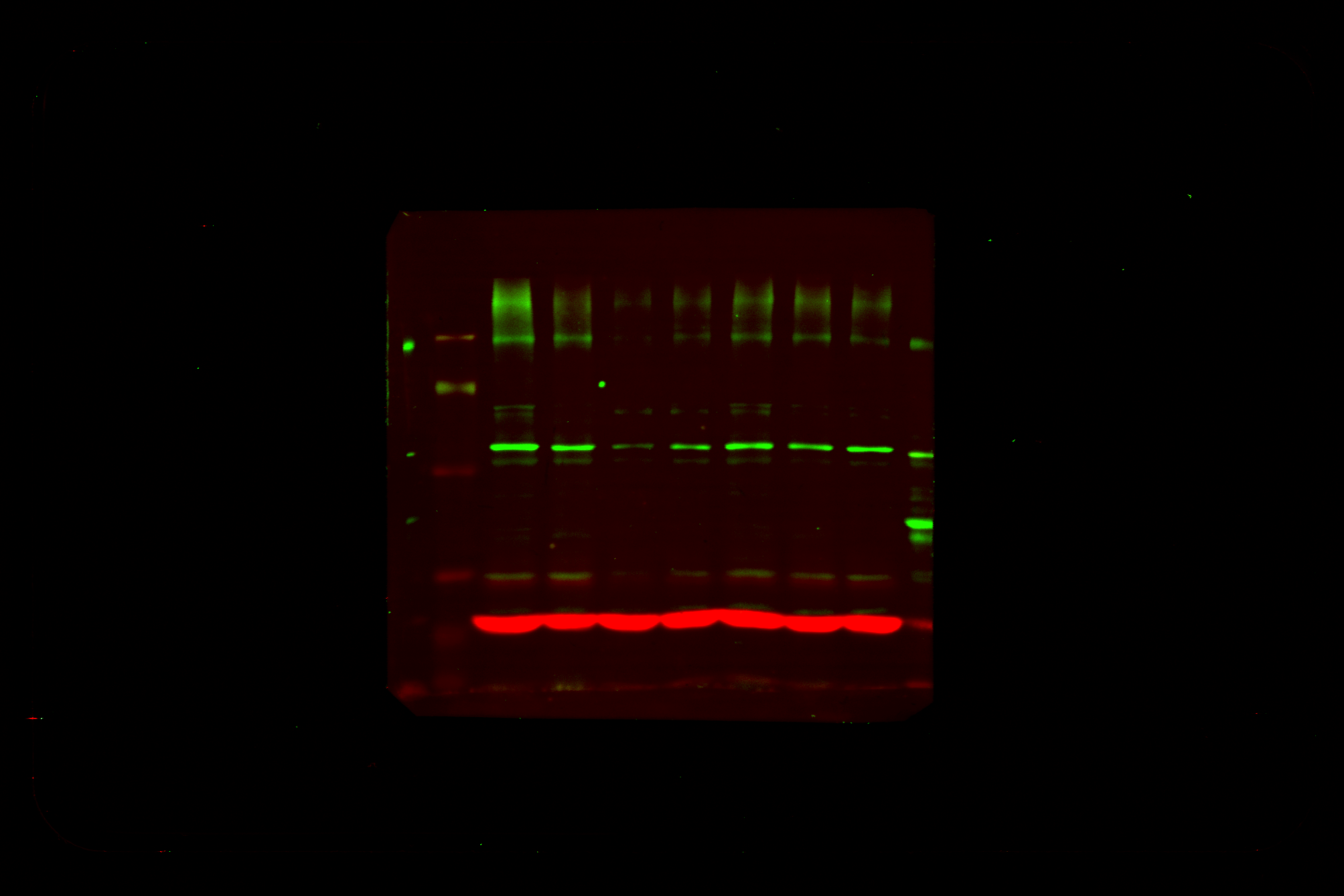

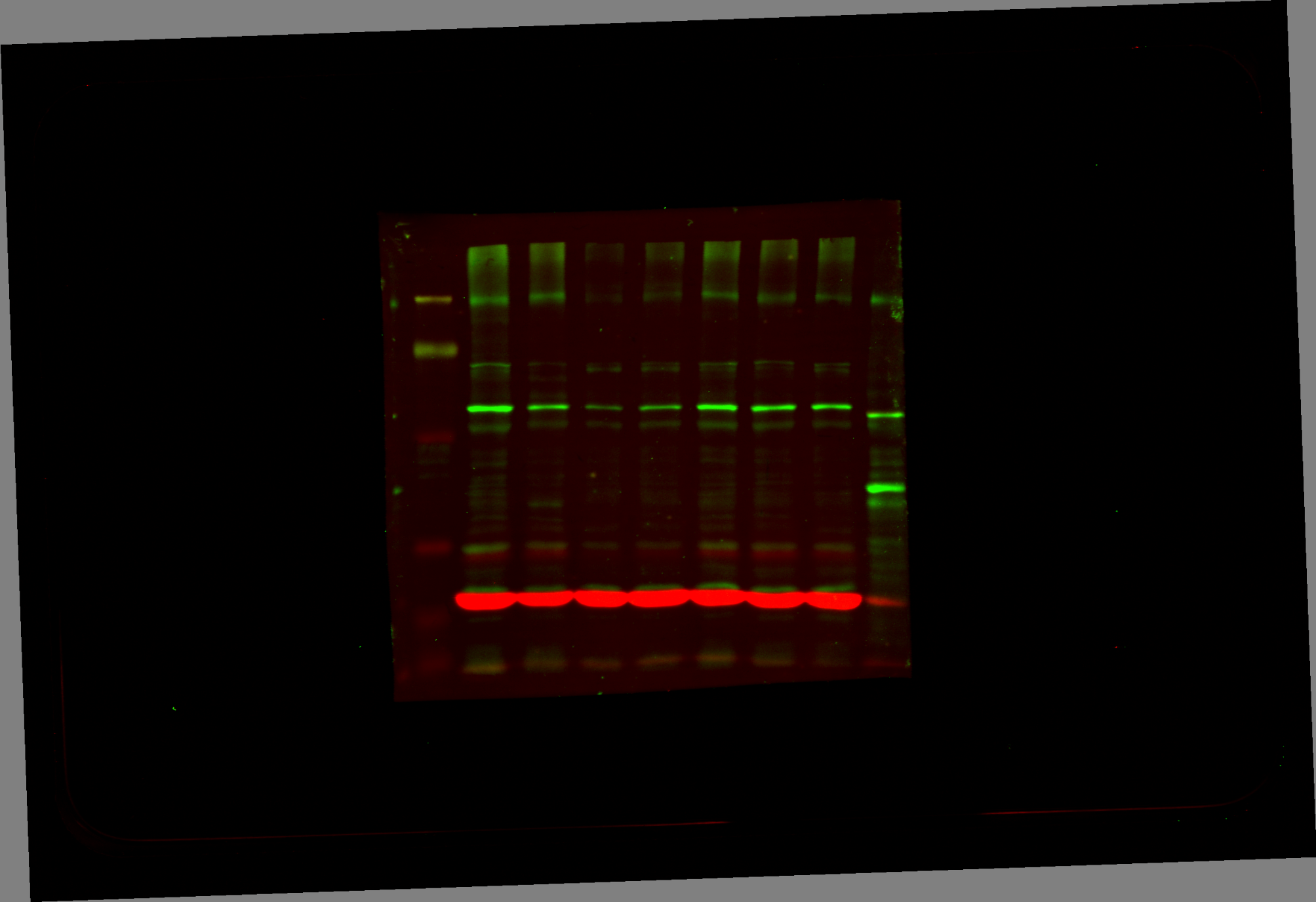
 **2 3 4**

ZO-1

β-actin

ZO-1

ZO-1

**
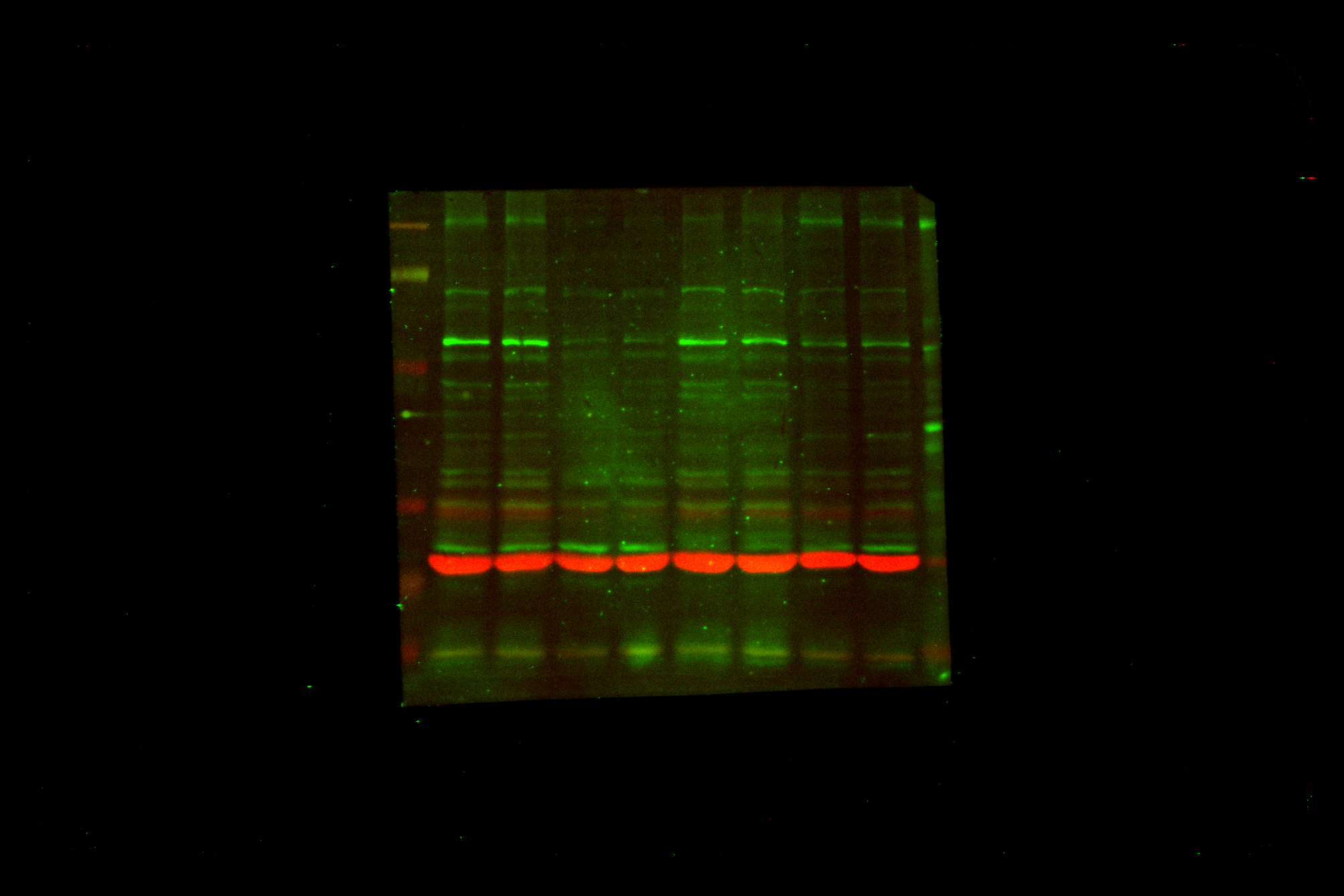
**

β-actin

ZO-1

**5**

β-actin

**6**

β-actin

ZO-1

The original full blots for E-cadherin in **Figure 4F**. Red boxes indicate the bands used in the figure.


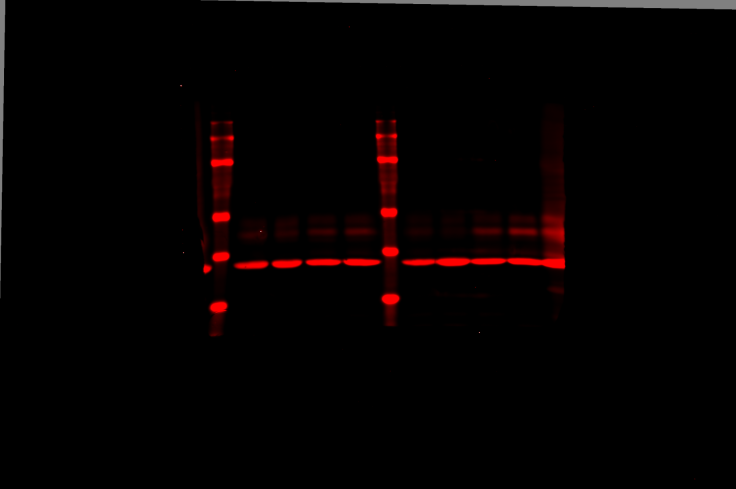


kDa

180

130

100

75

60

43

35

β-actin


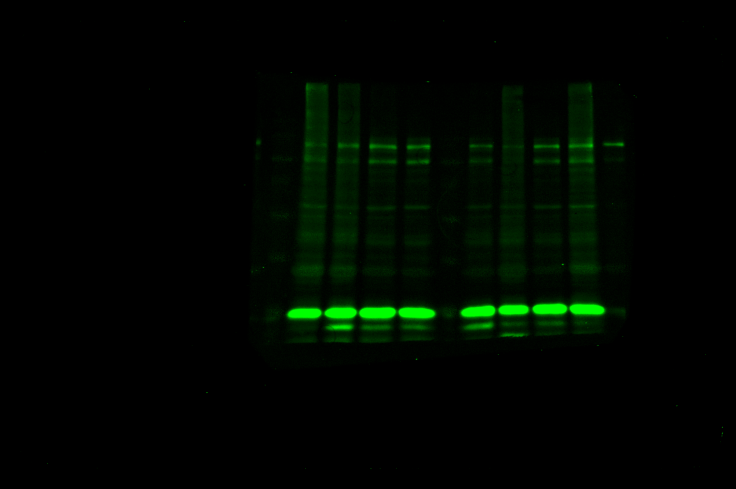
**1**

Veh

Il-1β

Scu

Scu+Il-1β

E-cadherin

ZO-1

β-actin


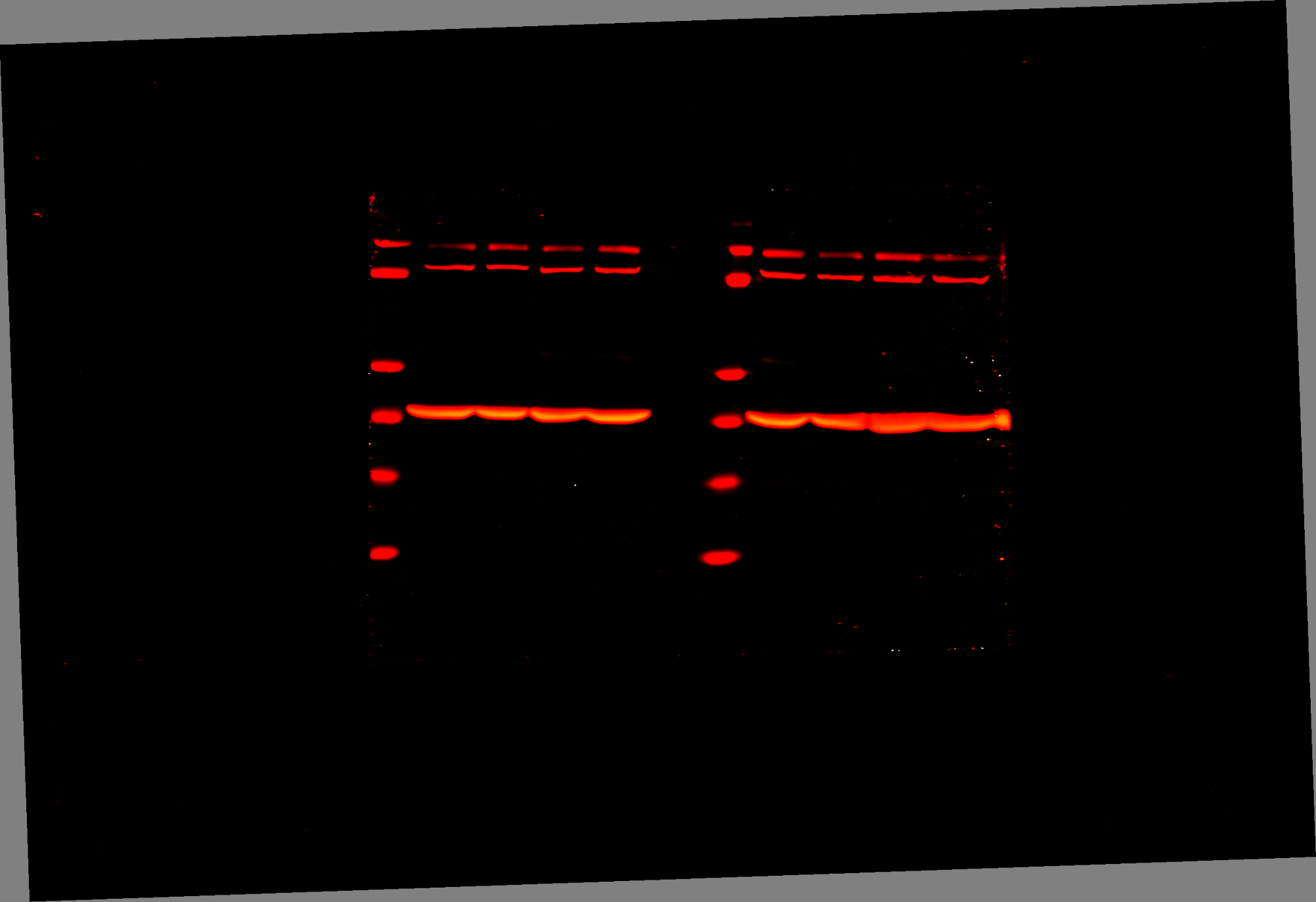

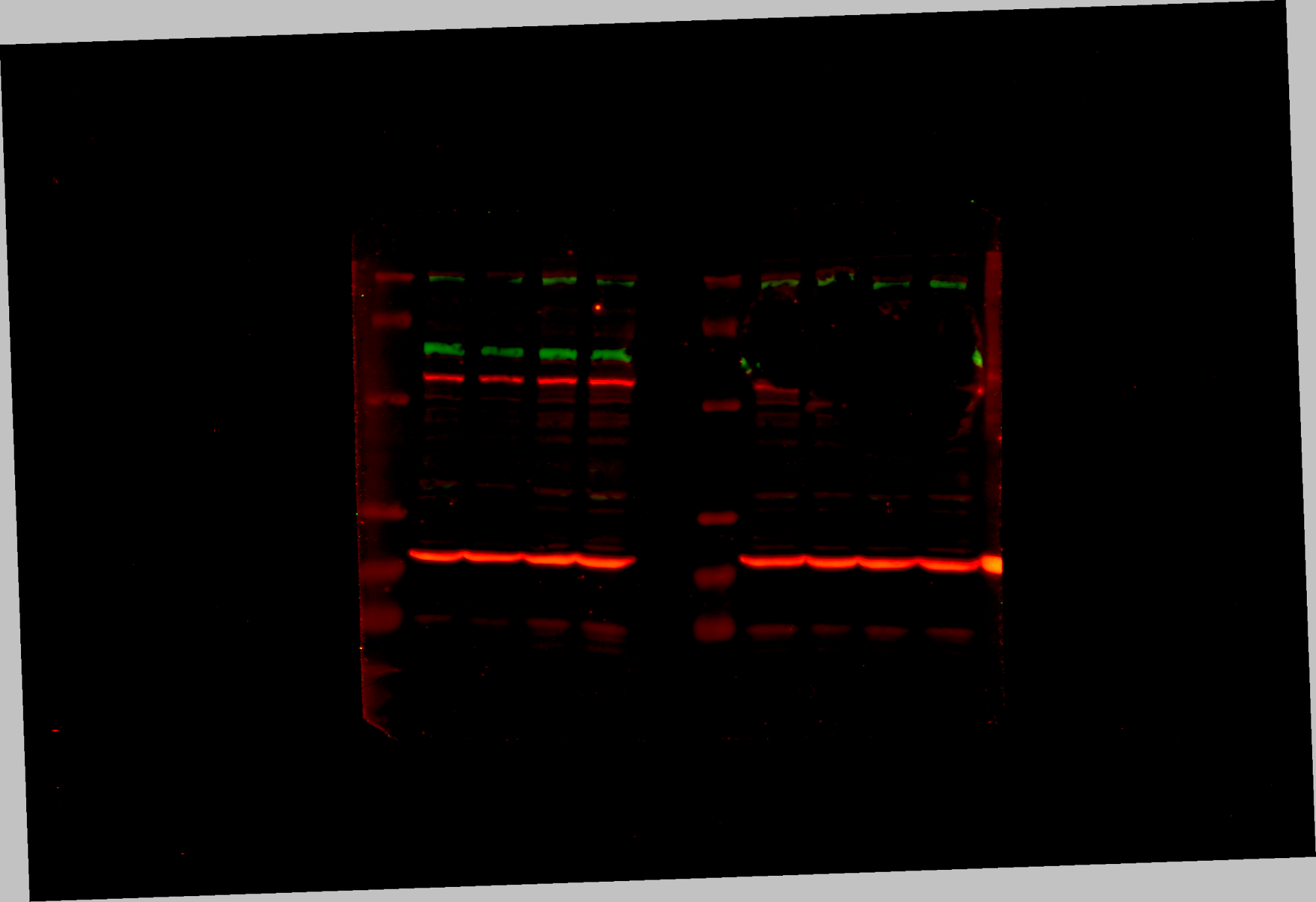


E-cadherin

**2-3**

E-cadherin

β-actin

β-actin


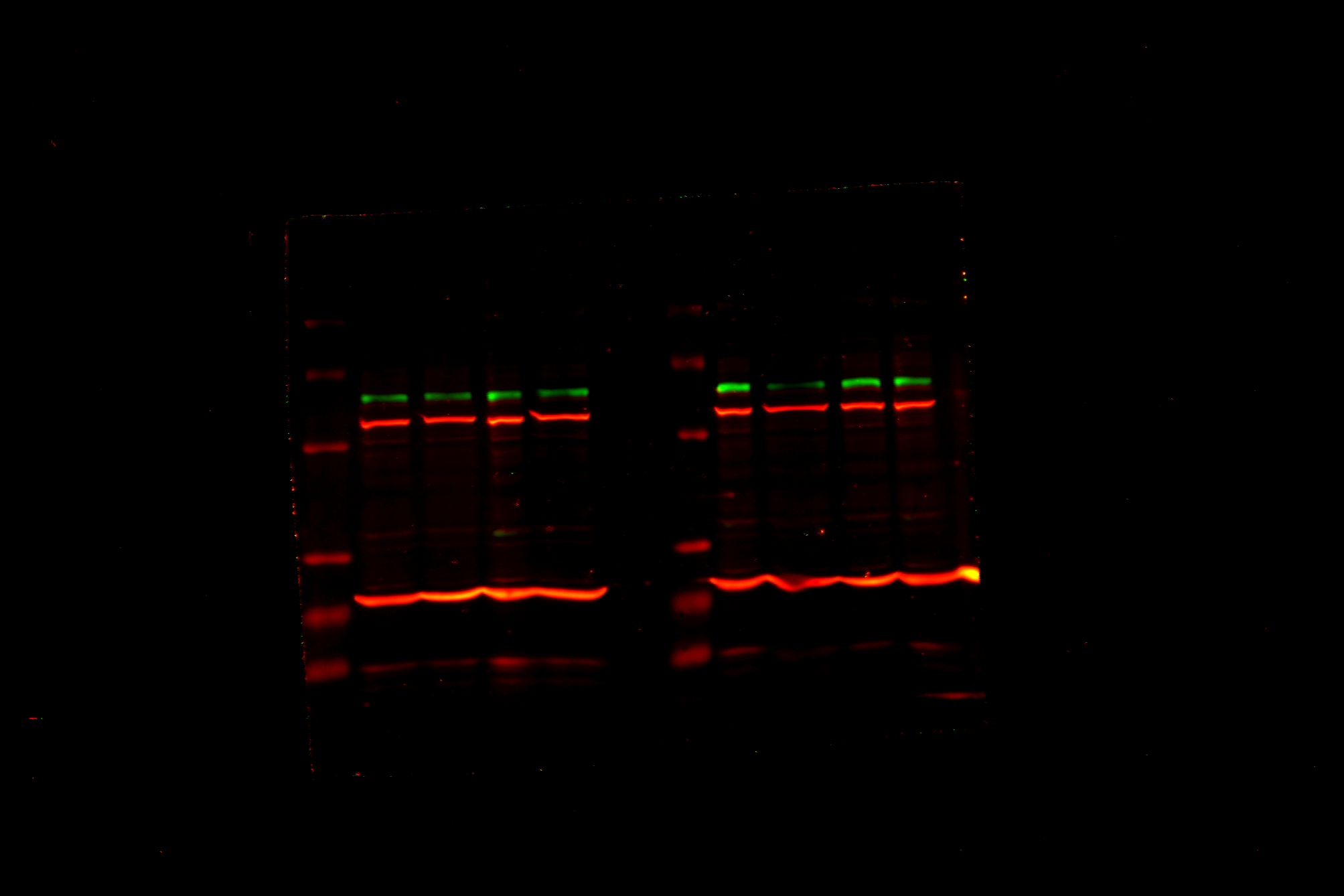

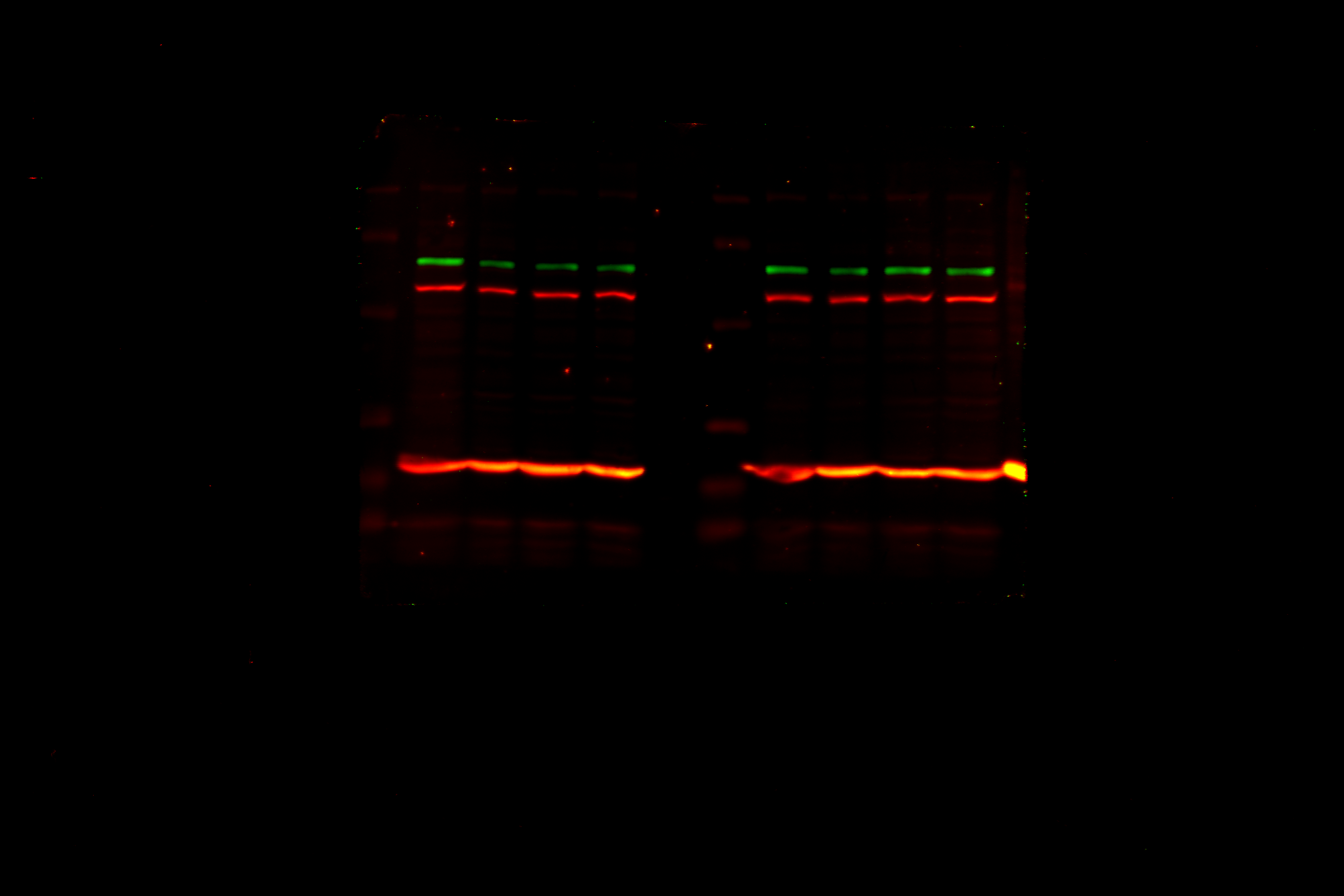
**4-5**

E-cadherin

β-actin

E-cadherin

β-actin

The original full blots for Occludin in **Figure 4F**. Red boxes indicate the bands used in the figure.

**1**

**
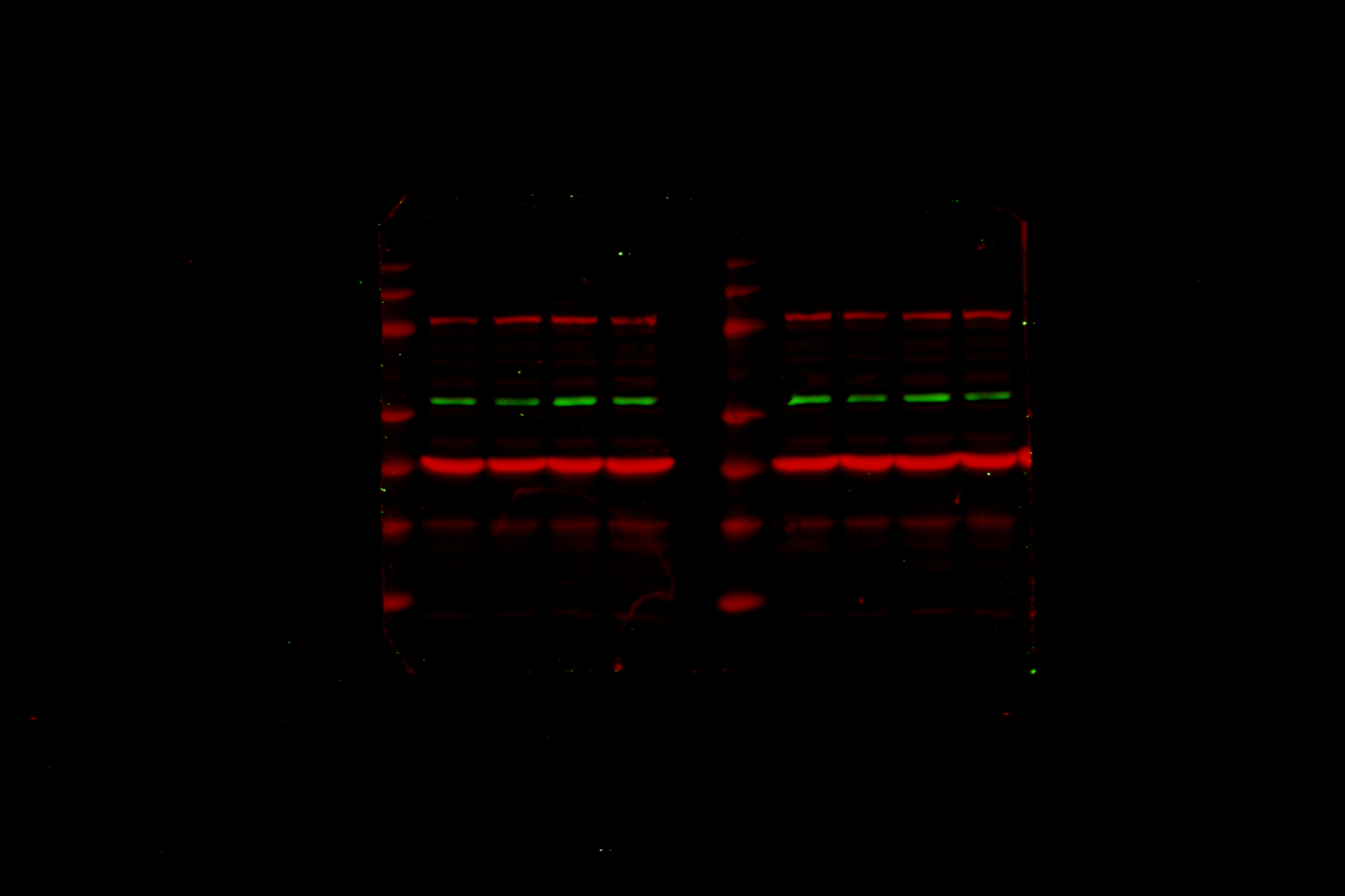
**

Veh

Il-1β

Scu

Scu+Il-1β

kDa

180

130

100

70

55

40

35

Veh

Il-1β

Scu

Scu+Il-1β

Occludin

β-actin

**2 3-4**

**
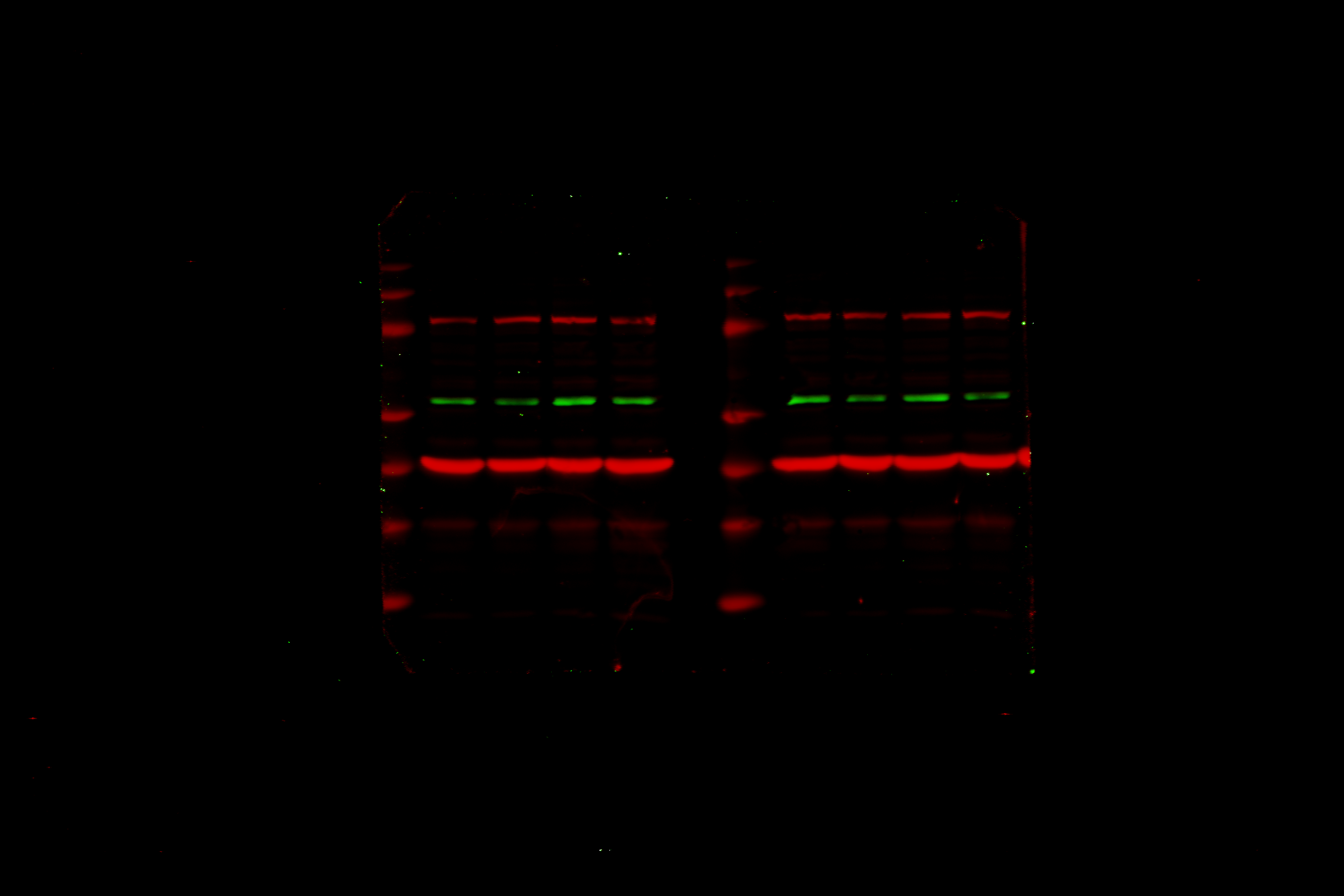

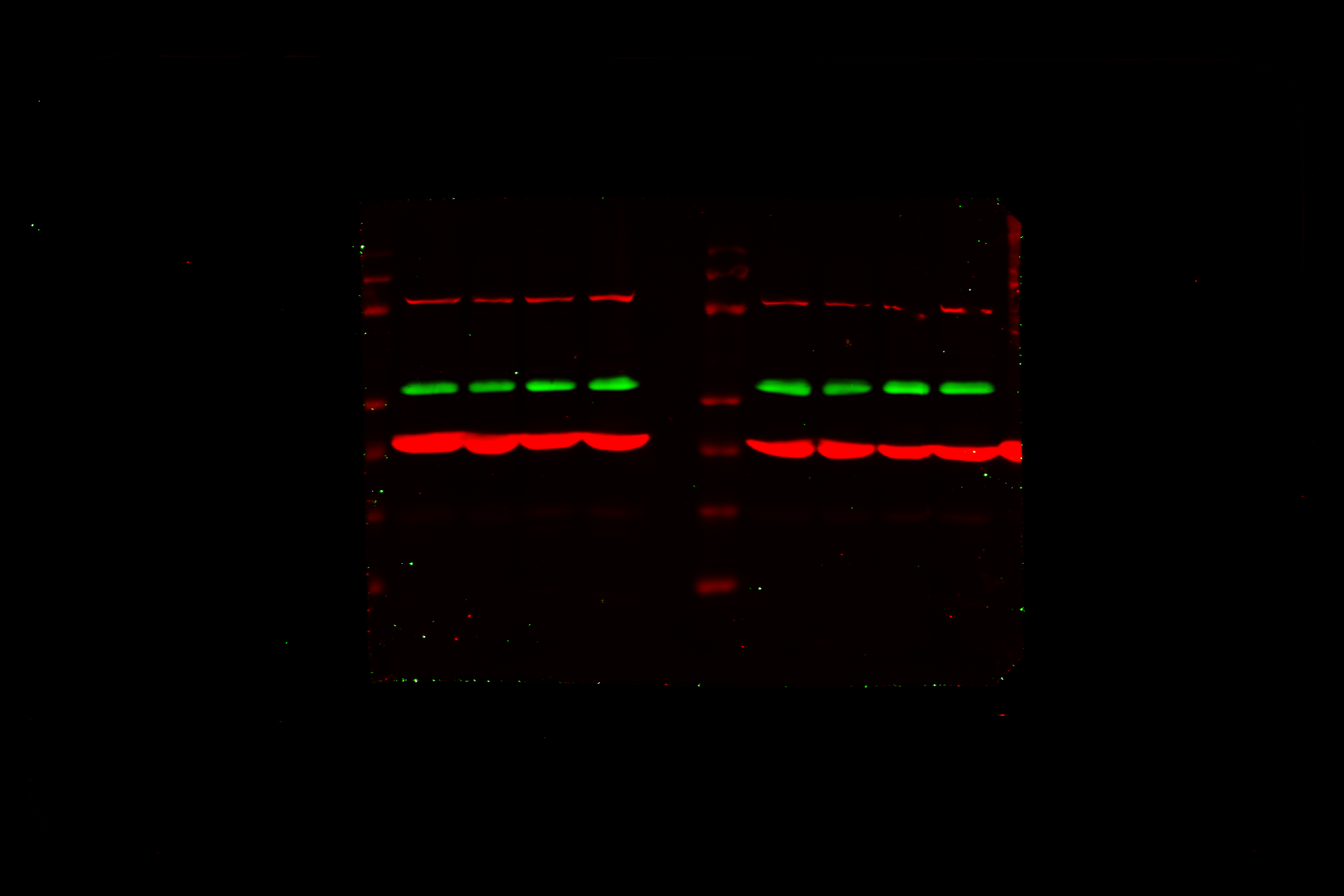
**

Occludin

β-actin

Occludin

β-actin

**5**

**
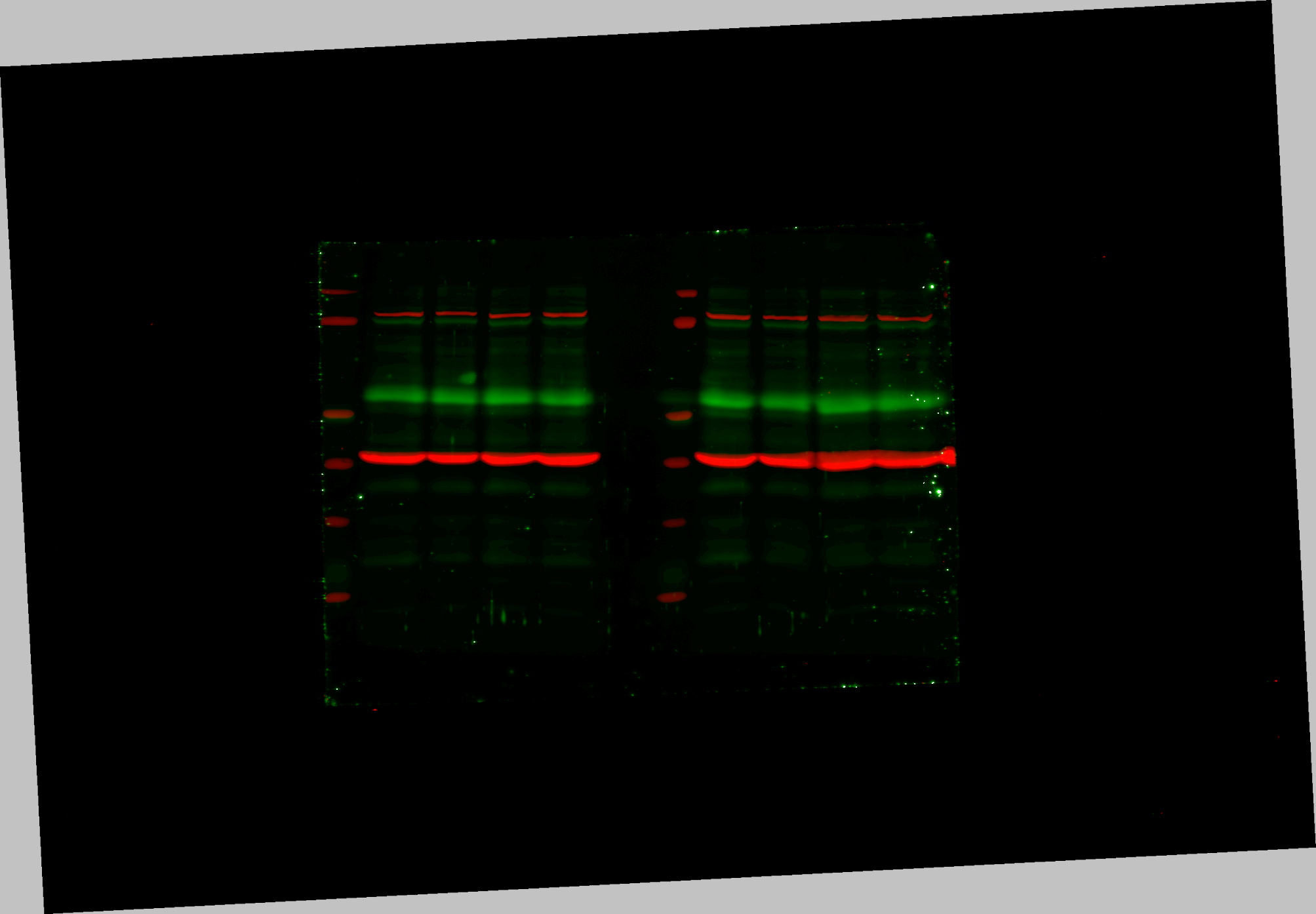
**

Occludin

β-actin

The original full blots for ZO-1 in **Figure 4F**. Red boxes indicate the bands used in the figure.

**
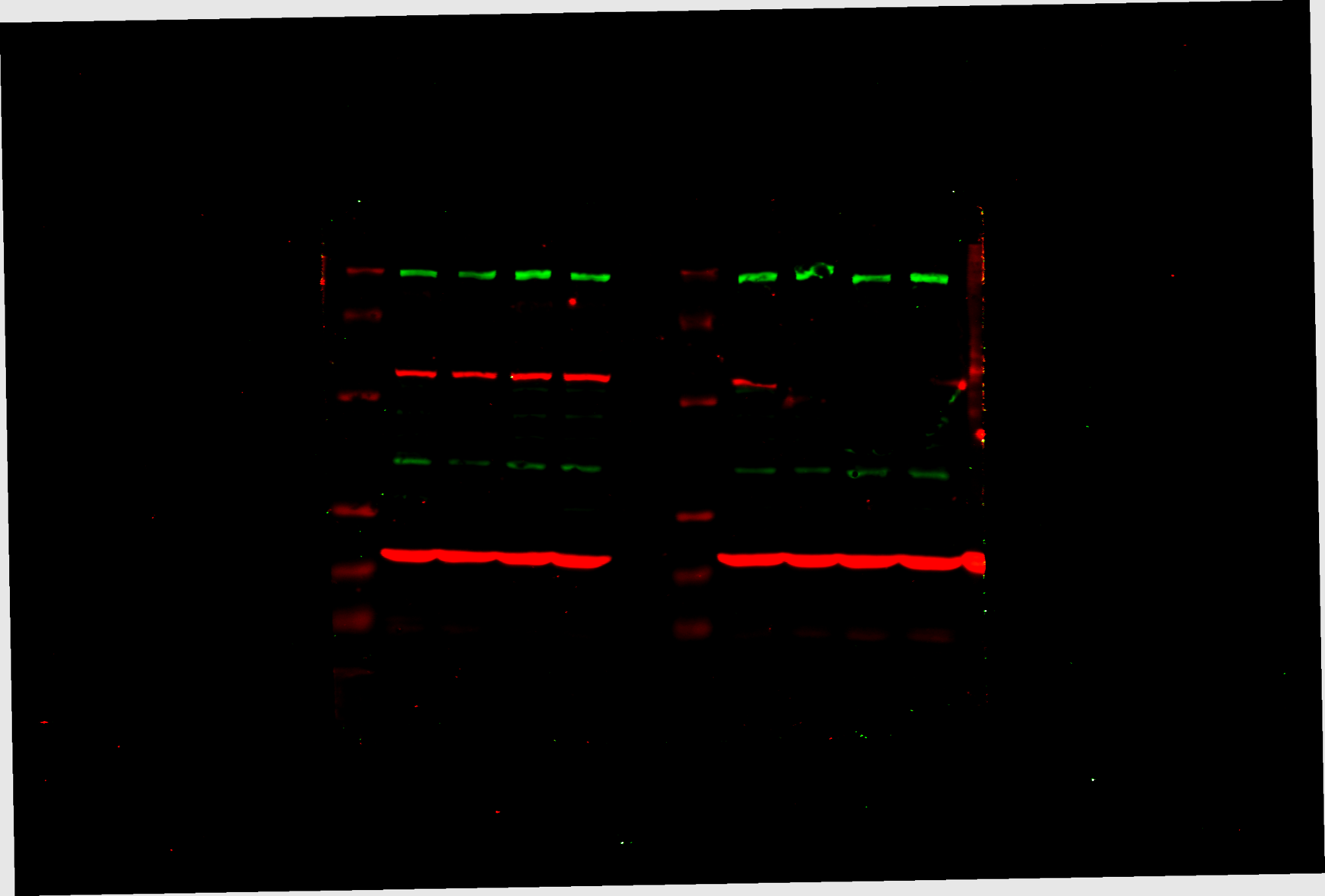

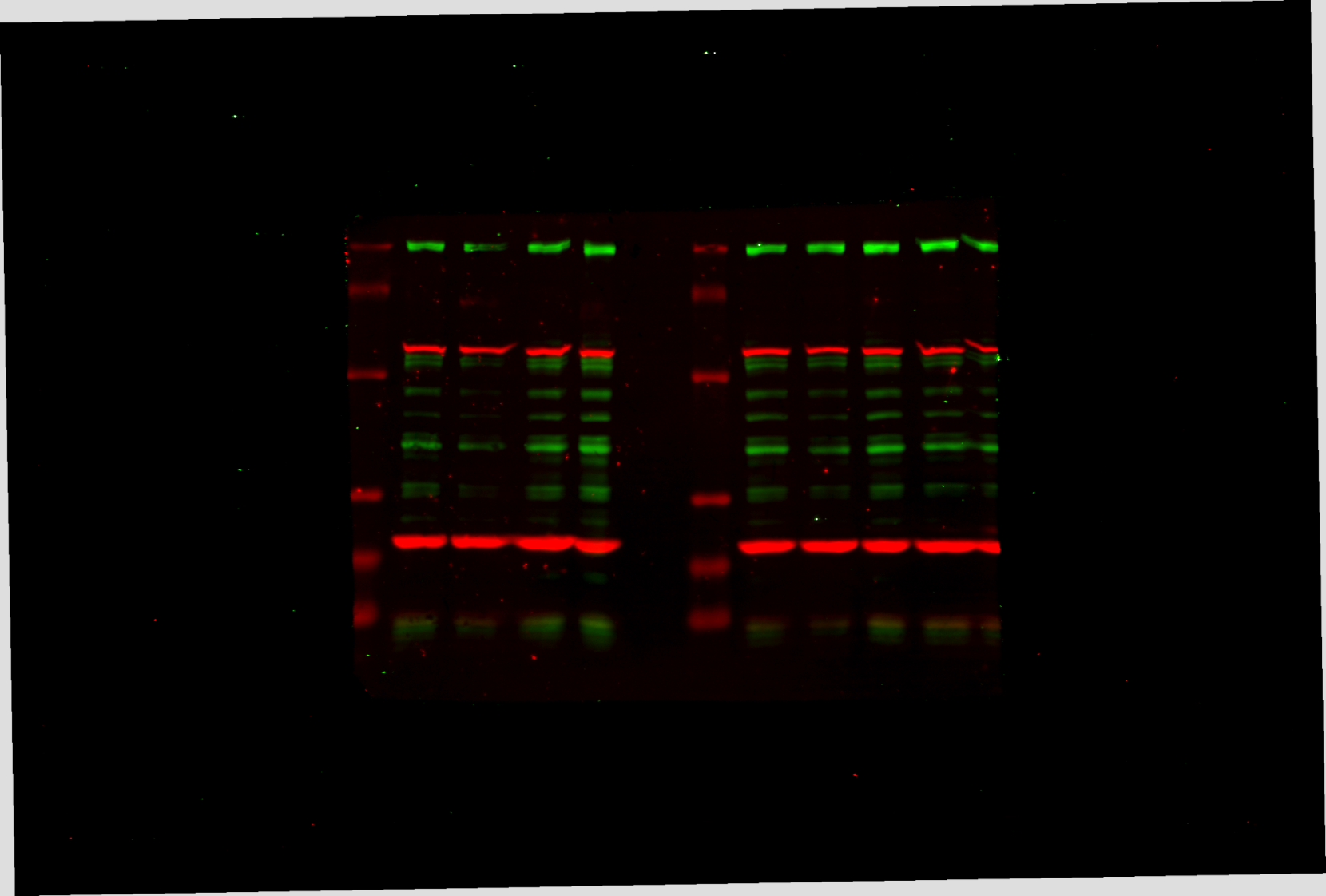
**

kDa

200

130

95

70

50

40

35

ZO-1

β-actin

Il-1β

Veh

Scu

Scu+Il-1β

ZO-1

β-actin


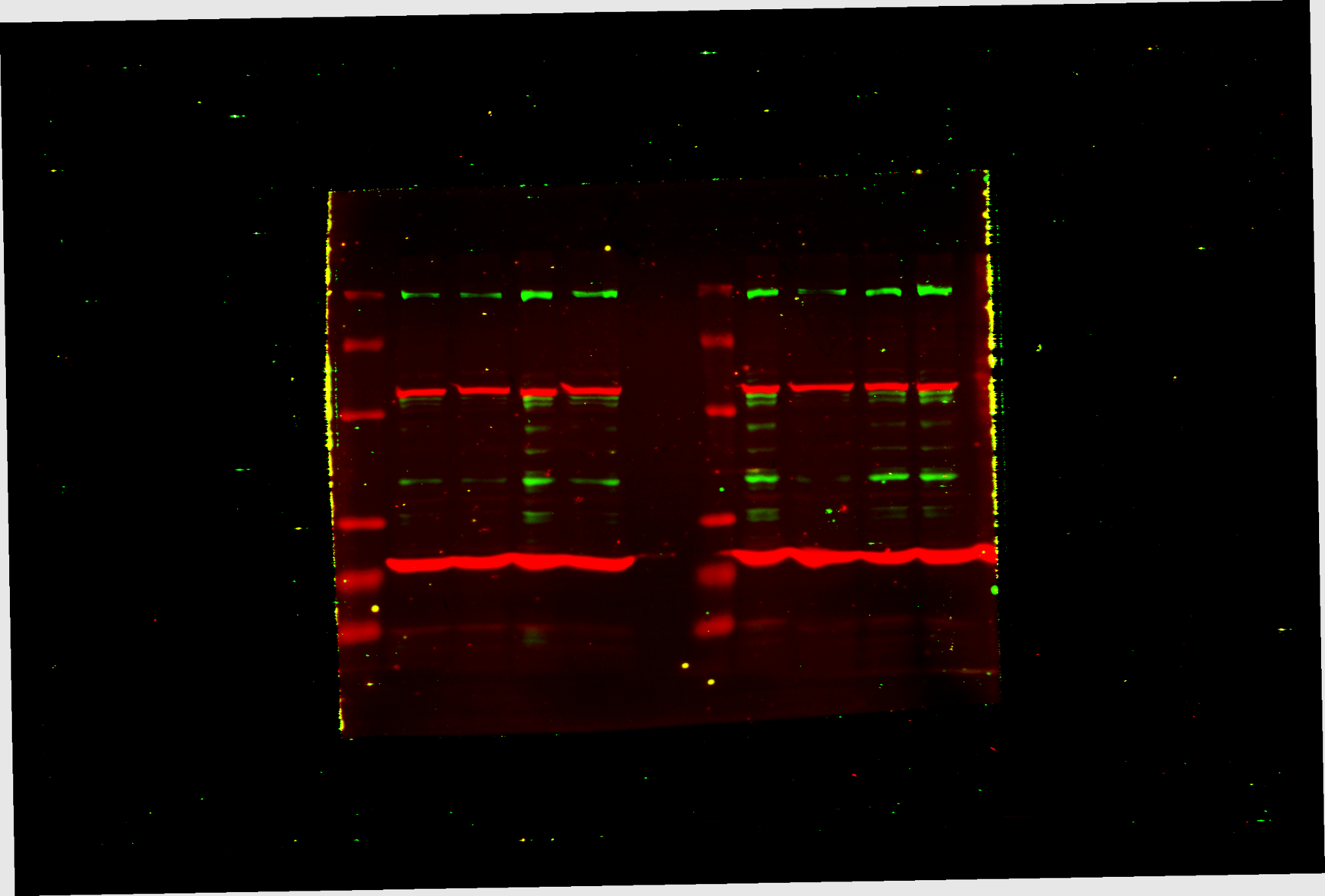


ZO-1

β-actin


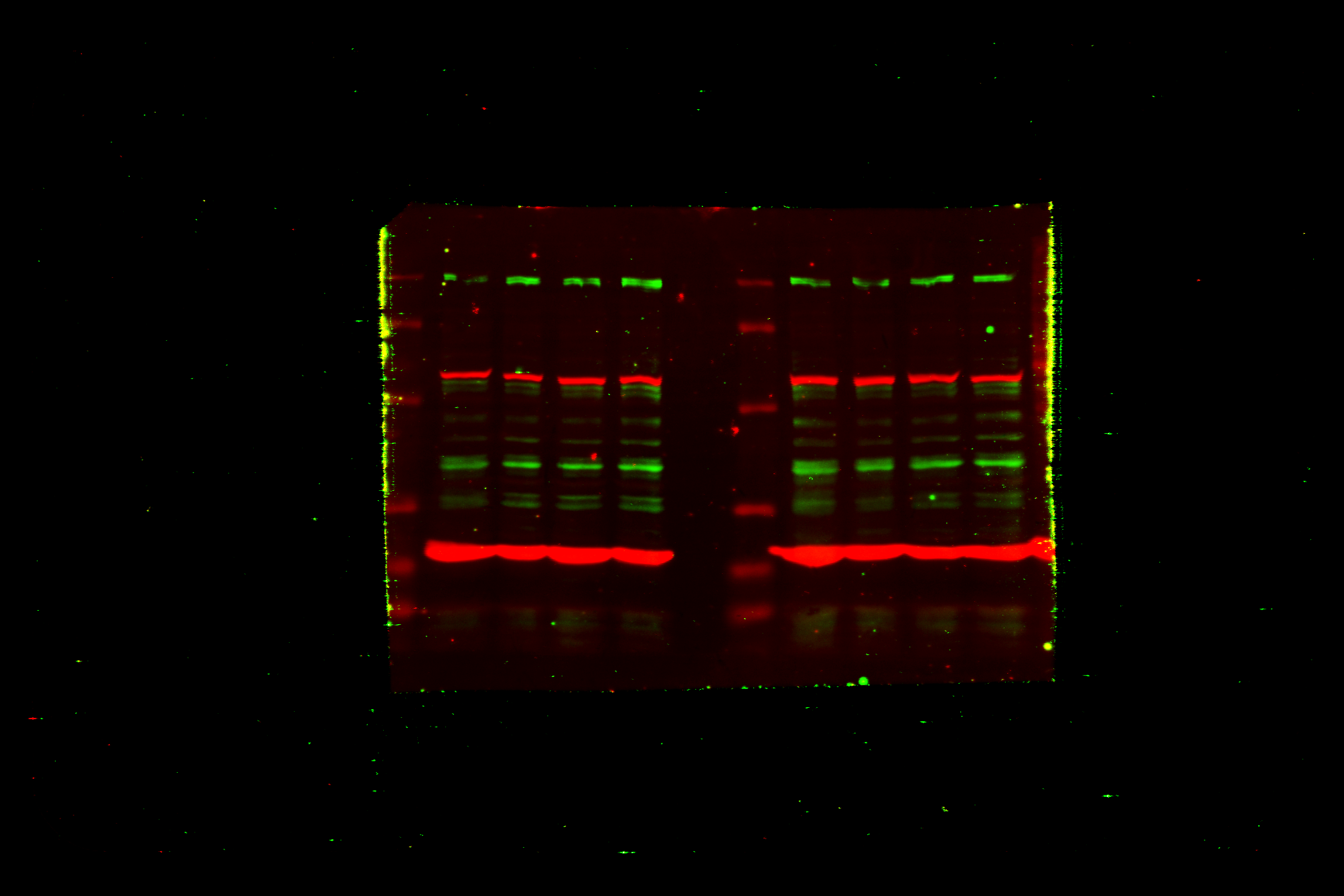


ZO-1

β-actin

The original full blots for P-NF-κB，NF-κB and GAPDH in **Figure 5A**. Red boxes indicate the bands used in the figure.


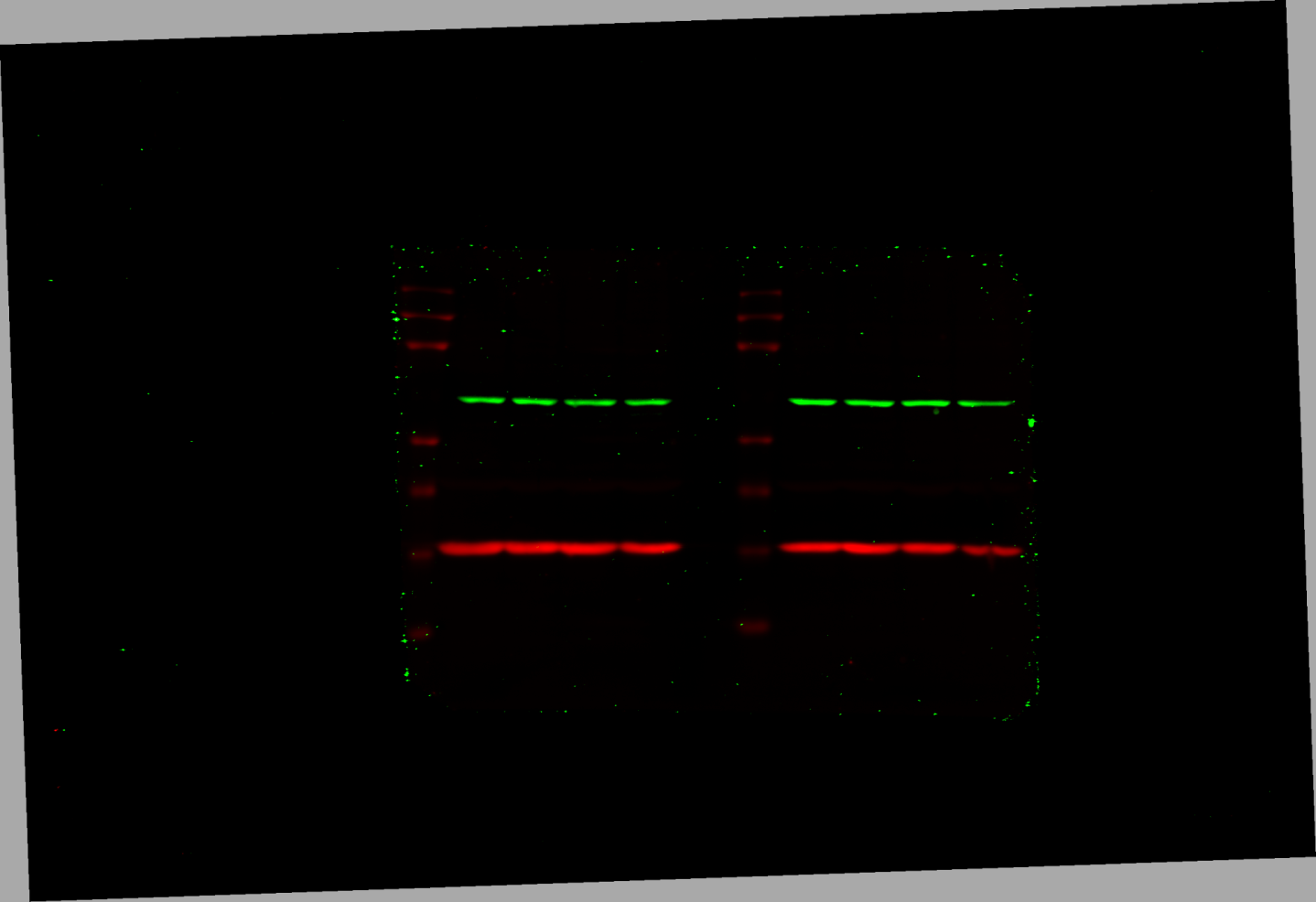


kDa

180

130

100

70

55

40

35


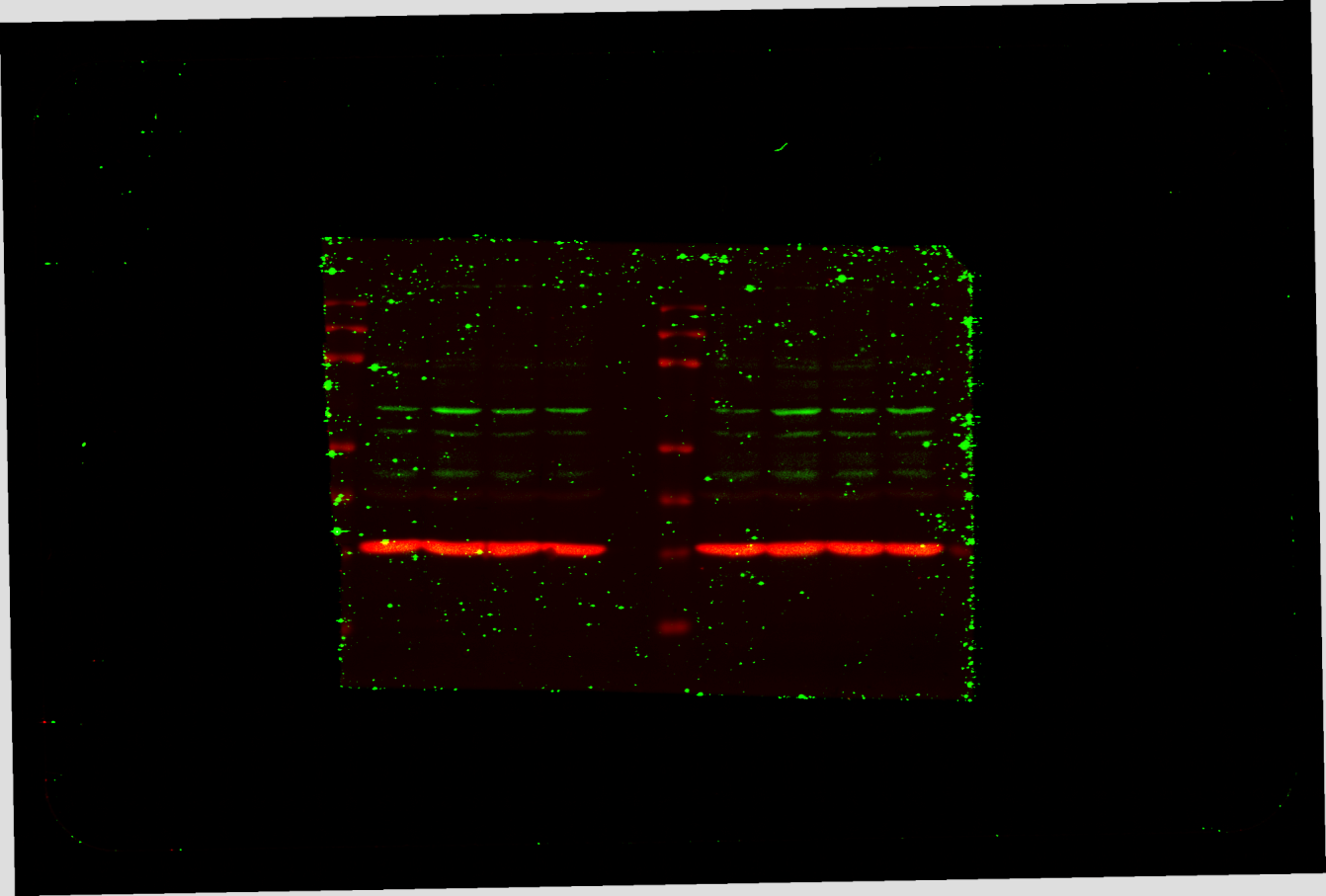


kDa

180

130

100

70

55

40

35

Veh

Il-1β

Scu

Scu+Il-1β

Veh

Il-1β

Scu

Scu+Il-1β

NF-κB

GAPDH

P-NF-κB

GAPDH

25


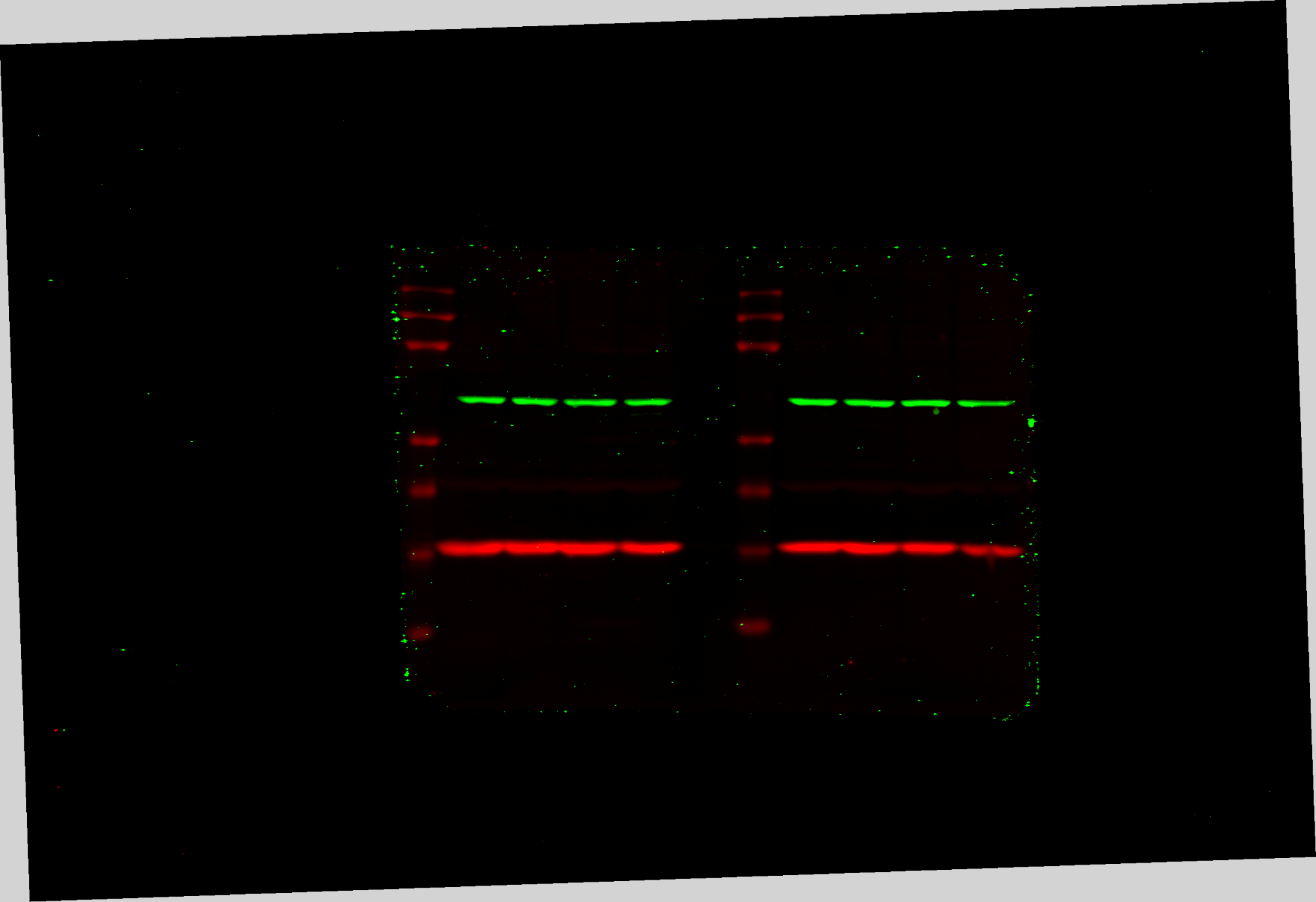

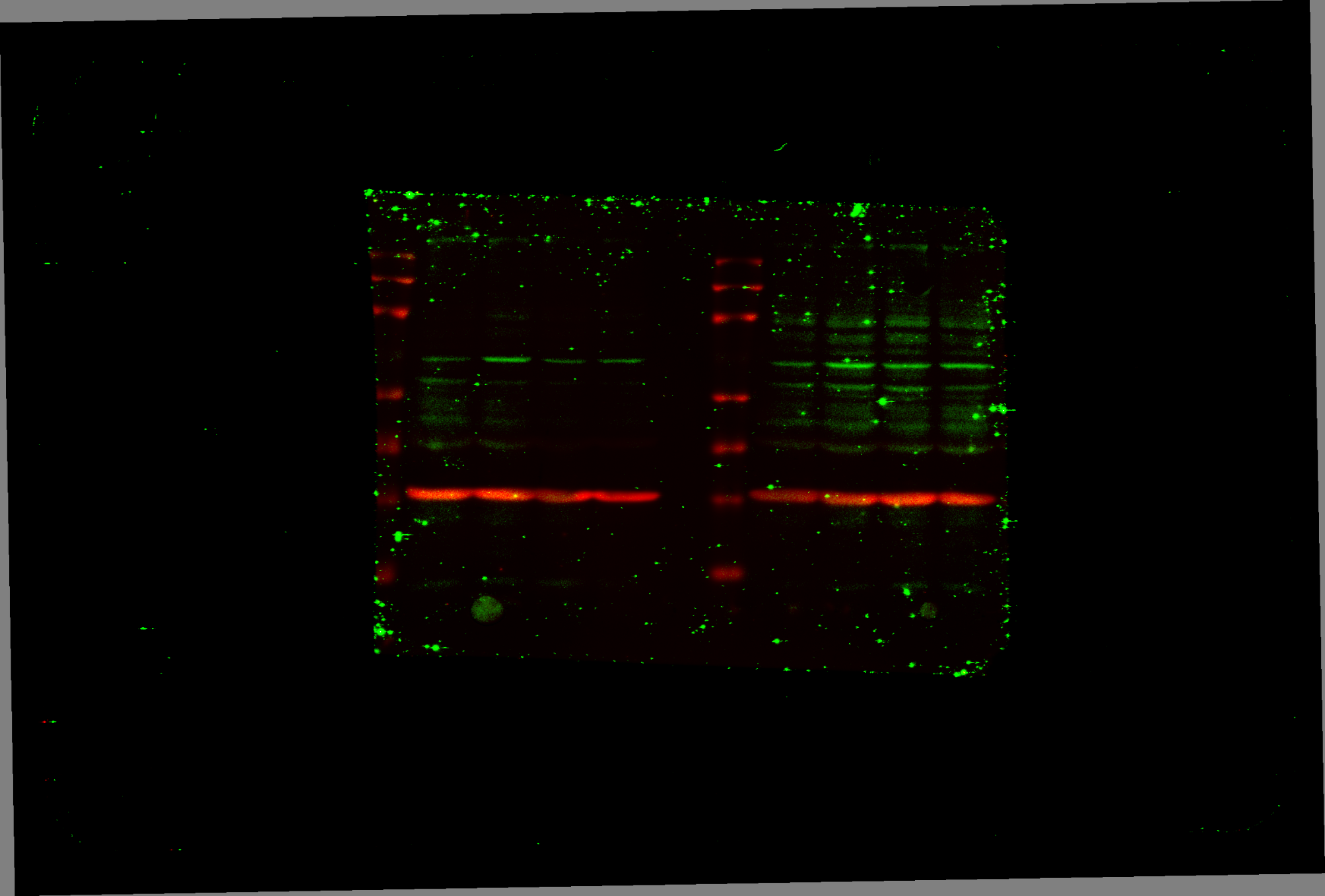


NF-κB

GAPDH

P-NF-κB

GAPDH


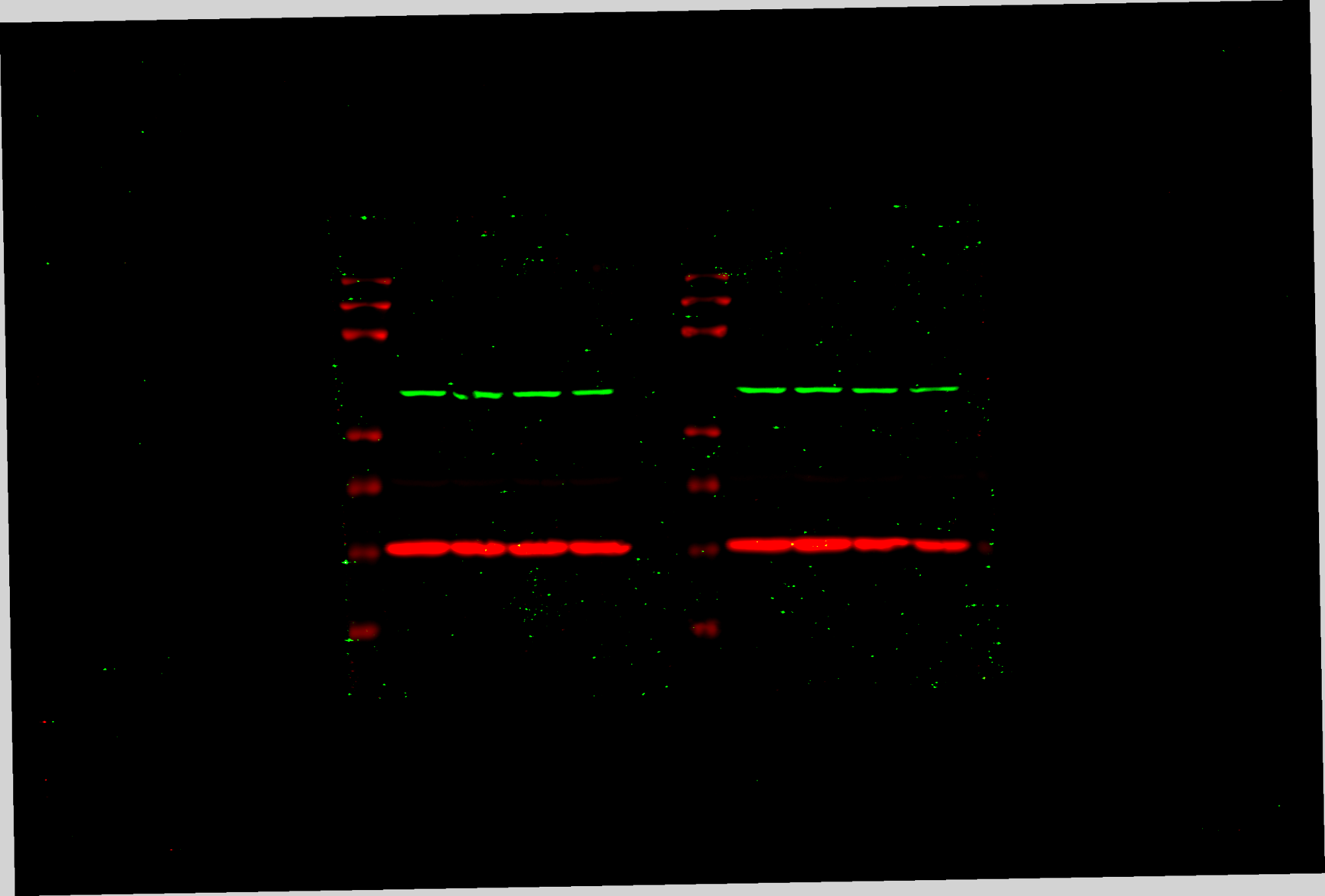

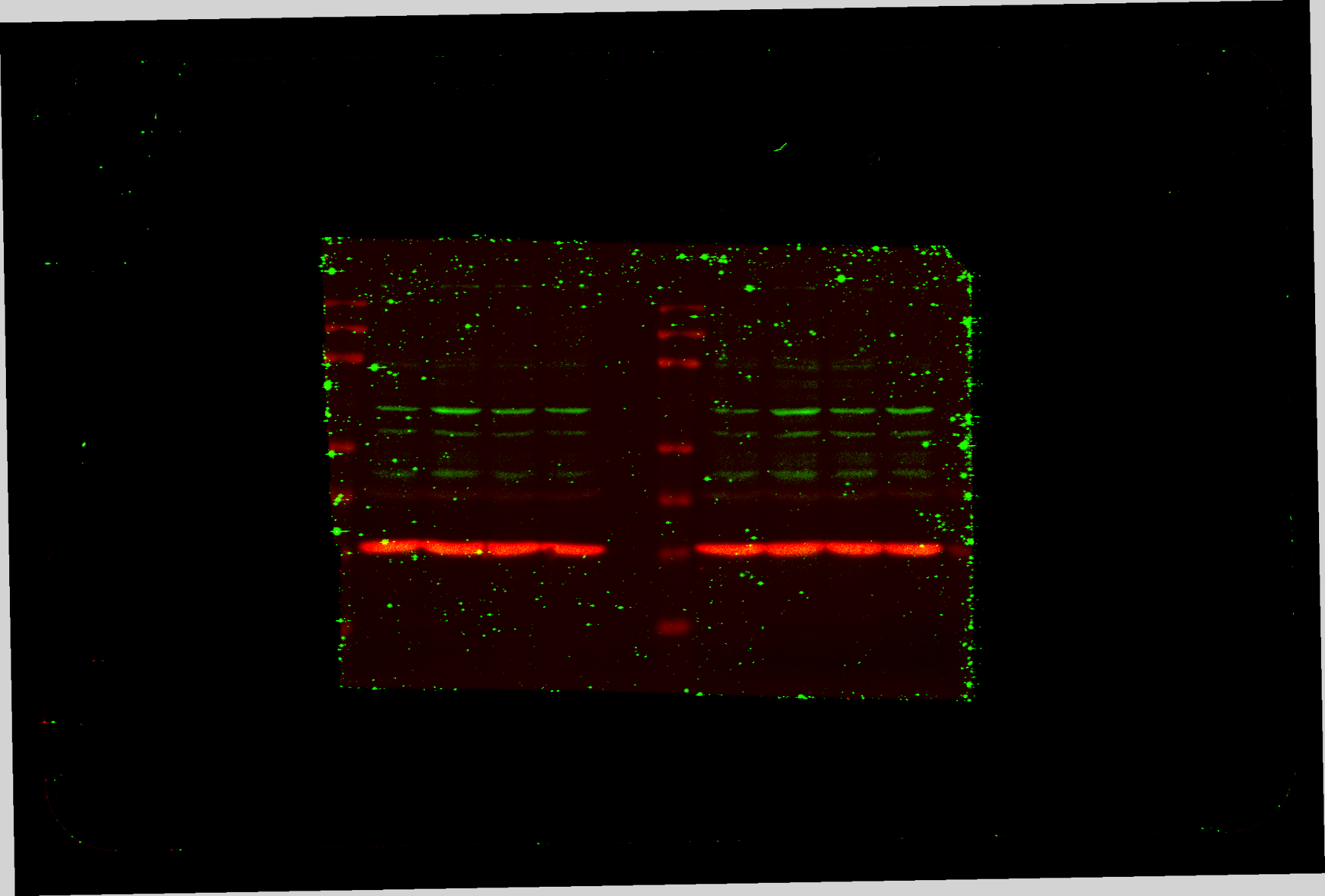


NF-κB

GAPDH

P-NF-κB

GAPDH
